# Molecular genetic basis and clinical heterogeneity of sitosterolemia: focusing on the mutation spectrum and pathogenic mechanisms of ABCG5/ABCG8 genes

**DOI:** 10.3389/fnut.2026.1857512

**Published:** 2026-07-17

**Authors:** Xiu Zhao, Zhe Su, Weimin Xiao, Chuanjun Liu, Rongfei Zheng, Zongwei Xu, Jinyu You, Guowu Yang, Yichun Wu, Wei Fang, Yuxin Cheng, Zhi Yu

**Affiliations:** 1 Department of Endocrinology, Shenzhen Children’s Hospital, Shenzhen University, Shenzhen, China; 2 Shenzhen SMQ Group Medical Laboratory, Shenzhen Academy of Metrology & Quality Inspection, Shenzhen, China; 3 School of Public Health (Shenzhen), Shenzhen Campus of Sun Yat-sen University, Sun Yat-Sen University, Shenzhen, China; 4 Medicine School, Shenzhen University, Shenzhen, China

**Keywords:** ABCG5 gene, ABCG8 gene, gene mutation, genetic heterogeneity, pathogenic mechanism, phytosterols, single heterozygous mutation, sitosterolemia

## Abstract

Sitosterolemia represents a rare autosomal recessive disorder of lipid metabolism, defined by the pathological accumulation of phytosterols—notably sitosterol and campesterol—in both plasma and tissues. This biochemical aberration precipitates severe clinical sequelae, including premature atherosclerosis, hemolytic anemia, and arthritis. The etiology is firmly established as loss-of-function mutations in *ABCG5* and *ABCG8*, which encode critical sterol efflux transporters. This review systematically dissects the molecular genetic architecture of sitosterolemia, offering a comprehensive evaluation of the *ABCG5/ABCG8* mutation spectrum. Our analysis encompasses canonical homozygous and compound heterozygous variants, while also addressing the emerging significance of monoallelic heterozygous mutations. We further examine how specific genotypic alterations impair transporter function and correlate with phenotypic severity. Moreover, by synthesizing recent findings, we investigate whether heterozygous carriers manifest subclinical traits characterized by incomplete penetrance and assess their associated pathogenic risks. Ultimately, this work establishes a robust theoretical framework to advance precise diagnosis, refine genetic counseling strategies, and facilitate personalized clinical management of sitosterolemia.

## 1 Introduction

Sitosterolemia (OMIM #210250), frequently referred to as phytosterolemia, represents a rare autosomal recessive disorder of lipid metabolism. This condition arises from biallelic loss-of-function variants within either the ATP-binding cassette subfamily G member 5 (*ABCG5*) or member 8 (*ABCG8*) loci (1, 2). Serving as an essential regulatory barrier, this transporter actively effluxes dietary phytosterols (such as sitosterol and campesterol) alongside cholesterol back into the gut lumen for fecal elimination or into bile for hepatic removal, thus restricting their systemic uptake and deposition (3). Defects in either gene impair the assembly or activity of this heterodimer, resulting in the disease’s defining biochemical signature: significantly elevated circulating plant sterol concentrations (4).

Clinically, sitosterolemia displays marked heterogeneity, often causing substantial diagnostic latency and frequent misclassification, particularly as familial hypercholesterolemia (FH) (2, 5). The phenotypic range spans from severe pediatric-onset pathology to attenuated adult presentations. Hallmark features include tendon and tuberous xanthomas, accelerated atherosclerosis precipitating premature cardiovascular events, and hematological disturbances such as macrothrombocytopenia and hemolytic anemia (6, 7). Nevertheless, the relationship between genotype and clinical severity remains intricate and largely unpredictable, with considerable variability noted even among subjects harboring identical mutations (1).

Although historically characterized by a recessive inheritance pattern necessitating biallelic mutations, emerging data are challenging this established genetic paradigm. A growing body of evidence indicates that individuals carrying a single pathogenic allele can display no symptoms while having mildly elevated plasma phytosterol levels, a condition characterized as “subclinical” sitosterolemia, and may face an increased cardiovascular risk compared to the general population (8, 9). These findings imply a more nuanced gene-dose effect, underscoring the potential influence of genetic modifiers or environmental factors on disease manifestation, thereby extending beyond the classical recessive model (9).

Consequently, this narrative review examines the molecular genetics of sitosterolemia, detailing the latest published mutational landscape of *ABCG5* and *ABCG8*, elucidating the pathogenic mechanisms involved, exhibiting the clinical features, diagnostic algorithm and treatment strategies, and discussing the evolving clinical implications of heterozygous variants and future directions.

## 2 ABCG5/ABCG8 biology and sterol transport

### 2.1 Gene locus, structure, and expression regulation

**Figure 1:**
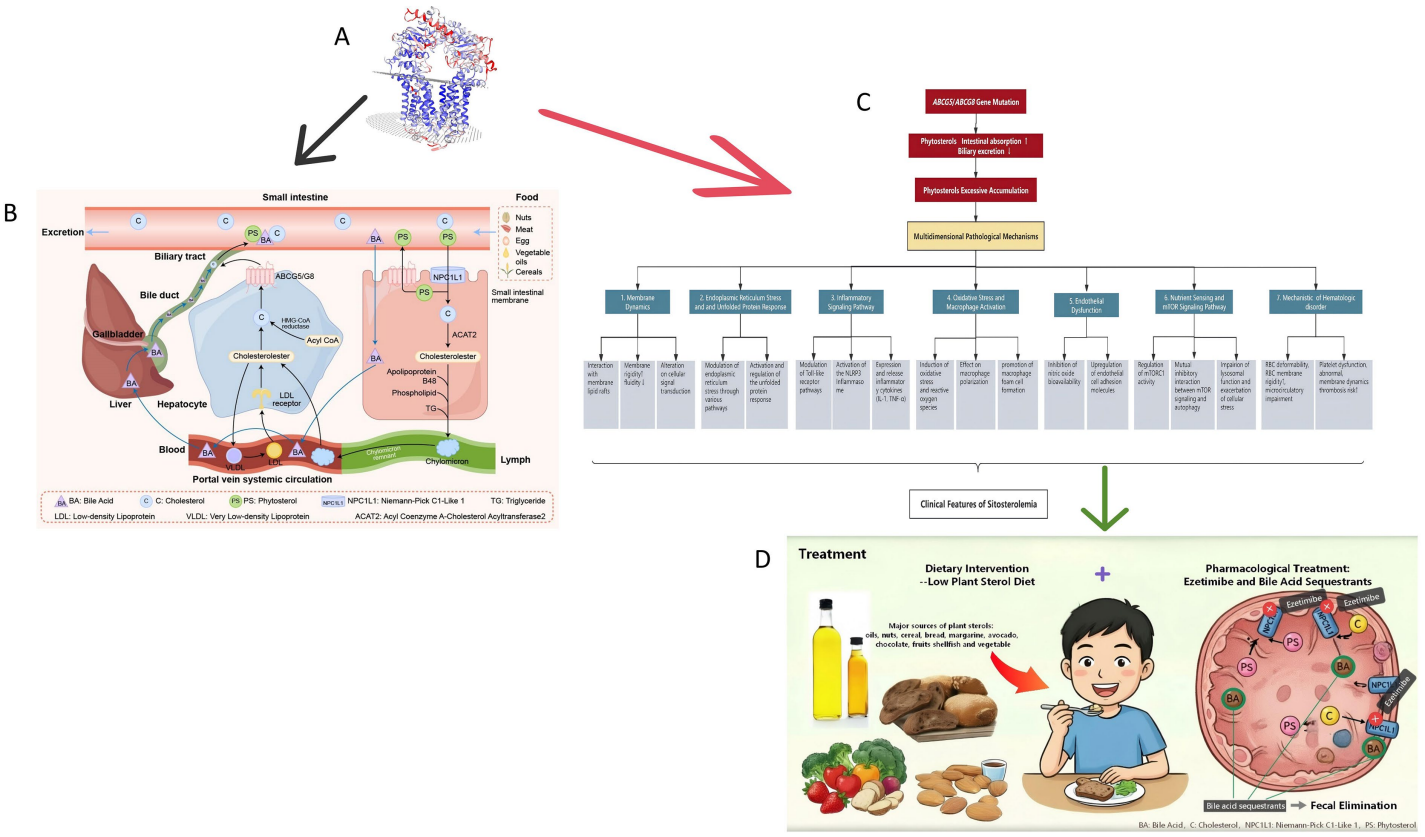
Ilustration summarizing *ABCG5*/*ABCG8* structure, sterol transport physiology, pathophysiological consequences, and therapeutic interventions in sitosterolemia. **(A)** The crystal structure of *ABCG5/ABCG8* (PDB accession number: 5DO7); **(B)** *ABCG5*/ABCG8 sterol transport pathway; **(C)** pathophysiological consequences in sitosterolemia; **(D)** therapeutic interventions for sitosterolemia.

**Figure 2:**
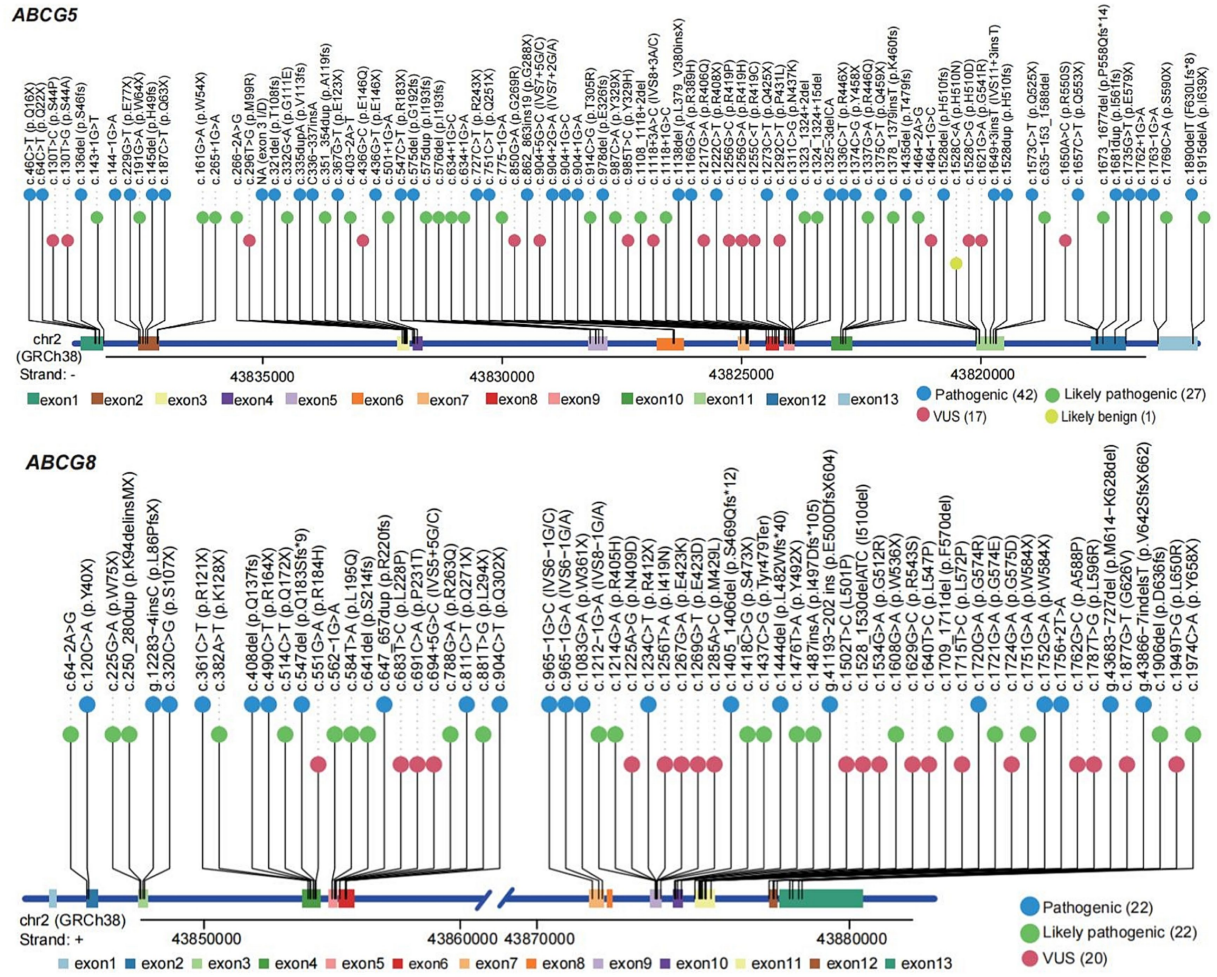
The locus and type of variants in the *ABCG5* and *ABCG8* genes associated with sitosterolemia. Based on the site mutation information of *ABCG5* and *ABCG8* documented in the disease databases Clinvar (https://www.ncbi.nlm.nih.gov/clinvar) and HGMD (https://www.hgmd.cf.ac.uk/ac), along with that in the literature (63). (1) The *ABCG5* mapping reveals 42 pathogenic loci, 27 likely pathogenic loci, 1 likely benign locus, and 17 variants of VUS within the dataset. (2) The mapping of ABCG8 uncovers 22 pathogenic loci, 22 likely pathogenic loci, and 20 variants of VUS in the dataset.

Positioned in a convergent head-to-head configuration on chromosome 2p21, the *ABCG5* and *ABCG8* loci exhibit a genomic architecture that underpins their synchronized transcriptional regulation and functional synergy (10). *ABCG5/ABCG*8 possess 13 exons, and their structures are presented in Figures 1A, 2.

This tandem arrangement is biologically imperative, given that both genes encode the respective subunits comprising a single heterodimeric transporter complex. Consequently, deleterious alterations in either locus impair the assembly of a competent transporter, thereby precipitating sitosterolemia, a rare autosomal recessive condition (1). Clinically, this disorder manifests through the excessive intestinal uptake of dietary phytosterols coupled with diminished biliary elimination, which drives their pathological deposition within plasma and peripheral tissues. Comprehensive genetic analyses have identified a broad array of pathogenic variants—encompassing nonsense, missense, frameshift, and splice-site defects across both genes—that account for the substantial phenotypic diversity observed among patients (2). Although *ABCG8* mutations reportedly predominate in certain demographics, investigations within Chinese cohorts suggest that *ABCG5* variants may be more frequent, highlighting distinct population-specific genetic landscapes (6).

### 2.2 Protein structure and heterodimer formation

*ABCG5* and *ABCG8* operate as half-transporters, each comprising one nucleotide-binding domain (NBD) and one transmembrane domain (TMD). To achieve functional competence, these subunits must assemble into a stable heterodimeric complex, commonly designated as sterolin-1 (ABCG5) paired with sterolin-2 (ABCG8), or simply G5G8 (11). This heterodimerization is indispensable for the correct trafficking of the complex to the plasma membranes of hepatocytes and enterocytes. Structural modeling and bioinformatics assessments of pathogenic missense variants, including the p.M99R substitution in *ABCG5*, reveal that such mutations compromise the tertiary architecture of the heterodimer, thereby disrupting its assembly and abolishing sterol transport efficiency (4). Furthermore, the preservation of a specific polar relay network within the TMDs is vital for allosteric modulation and ATPase activity; perturbations in this network, exemplified by the R543S variant, severely diminish the transporter’s catalytic performance (12). Consequently, disease-associated mutations primarily exert their effects by obstructing the formation or destabilizing the *ABCG5/G8* heterodimer, which constitutes the essential functional unit.

### 2.3 Physiological function: dual mechanism of intestinal absorption inhibition and hepatobiliary excretion promotion

The *ABCG5/G8* heterodimer plays a pivotal dual function in maintaining sterol homeostasis by restricting intestinal uptake and enhancing the biliary elimination of neutral sterols, such as cholesterol and phytosterols (10). Within small intestinal enterocytes, this transporter actively effluxes absorbed sterols back into the gut lumen, consequently diminishing their net absorption. Concurrently, in hepatocytes, it mediates the secretion of sterols into bile. This synergistic mechanism constitutes the body’s principal defense against the buildup of non-cholesterol sterols. Loss-of-function mutations in either *ABCG5* or *ABCG8* eliminate this efflux capacity, resulting in the defining features of sitosterolemia: excessive intestinal absorption and impaired biliary excretion of phytosterols, and occasionally cholesterol (13). The subsequent systemic phytosterols cumulation underlies the clinical phenotype, which spans from xanthomas and premature atherosclerosis to hematological disorders including macrothrombocytopenia and hemolytic anemia (7). The specificity of this transporter is highlighted by its essential role in selectively preventing the absorption of phytosterols, which are typically minimally absorbed in humans. ABCG5/ABCG8 sterol transport pathway is shown in Figure 1B.

### 2.4 Pathophysiological consequences of *ABCG5*/*ABCG8* dysfunction in sitosterolemia

Sitosterolemia, caused by loss-of-function mutations in the *ABCG5* or *ABCG8* genes, leads to increased intestinal absorption and decreased biliary excretion of phytosterols, resulting in their excessive accumulation in plasma and various tissues. Despite the structural similarity between phytosterols and cholesterol, their metabolic pathways and biological effects differ significantly, contributing to the complex pathophysiology of sitosterolemia (14, 15).

From a pathophysiological standpoint, the accumulation of phytosterols influences multiple biological systems via diverse mechanisms and contributes to vascular damage and a high risk of atherosclerotic cardiovascular disease (ASCVD). At the cellular membrane level, phytosterols, structurally similar to cholesterol, integrate into plasma membranes and preferentially associate with lipid rafts—specialized microdomains enriched in sterols and sphingolipids (16). Phytosterols play a pivotal role in maintaining membrane homeostasis and facilitating cellular processes dependent on lipid raft integrity. The incorporation of phytosterols into lipid bilayers alters membrane physical properties by increasing order and packing density, thereby enhancing membrane rigidity and reducing fluidity. Alterations in membrane dynamics due to phytosterol cumulation. These changes have significant consequences for cell signaling pathways and may influence diverse cellular responses, including immune signaling and stress adaptation. Phytosterols, such as *β*-sitosterol and other phytosterols, have been implicated in modulating endoplasmic reticulum (ER) stress through various pathways involves activation of classical unfolded protein response (UPR) sensors and downstream effectors, modulating cellular homeostasis and apoptosis (17–19). Phytosterols, such as β-sitosterol and stigmasterol, have been shown to activate Toll-like receptor (TLR) pathways and NLRP3 inflammasome by interfering with both upstream signaling and inflammasome assembly (20–23). These lead to excessive production and release of inflammatory factors such as interleukin-1 (IL-1) and tumor necrosis factor-alpha (TNF-*α*) (24, 25). Abnormal high levels of phytosterols can induce oxidative stress through increased generation of reactive oxygen species (ROS) (19, 26). This ROS generation is critical in modulating macrophage polarization and inflammatory signaling, establishing a link between phytosterol cumulation and oxidative stress-mediated macrophage activation (27, 28). Macrophage foam cell formation is a hallmark of atherosclerosis, driven by the uptake and accumulation of modified lipids, including oxidized low-density lipoprotein and phytosterols through multiple mechanism (26, 29, 30). Phytosterols, particularly elevated in conditions like sitosterolemia, have been implicated in impairing endothelial nitric oxide (NO) bioavailability (31, 32). Endothelial dysfunction in the context of elevated phytosterols also involves the upregulation of endothelial cell adhesion molecules such as VCAM-1 and ICAM-1, which facilitate leukocyte adhesion and transmigration, promoting vascular inflammation (31, 33). The impaired NO bioavailability and increased adhesion molecule expression promote endothelial activation, facilitating lipid infiltration and inflammatory cell recruitment into the arterial intima. In sitosterolemia, the excessive deposition of phytosterols in vascular tissues accelerates these processes, leading to premature and rapidly progressive atherosclerosis (25, 34, 35). Furthermore, phytosterols, such as stigmasterol and *β*-sitosterol, can act as nutrient signals that regulate mTORC1 activity, integrating sterol metabolism with cellular nutrient sensing and anabolic signaling (36–39). For instance, stigmasterol activates the mTOR signaling pathway by stabilizing oxysterol binding protein 5 (ORP5), which recruits mTOR to the lysosomal surface, promoting mTORC1 activation and enhancing biosynthetic processes like milk synthesis in bovine mammary epithelial cells (40). The mTOR pathway is a well-known inhibitor of autophagy. Phytosterols have been implicated in Mutual inhibitory interaction between mTOR signaling and autophagy (39). Disruptions in lipid metabolism such as sitosterolemia affects mTOR-dependent signaling pathways by modifying cellular lipid composition and nutrient availability (41). mTORC1 localizes to the lysosomal membrane, where it senses nutrient status and regulates lysosomal biogenesis and function. Phytosterols, such as stigmasterol, facilitate mTOR recruitment to lysosomes by stabilizing ORP5, enhancing mTOR signaling and promoting anabolic processes (40). Additionally, mTOR signaling modulates cellular stress responses by integrating signals from nutrient availability and lipid metabolism, thereby influencing autophagy and lysosomal degradation pathways.

In the hematologic system, phytosterols, which accumulate excessively in sitosterolemia due to ABCG5/ABCG8 mutations, integrate into red blood cell (RBC) membranes, altering their lipid composition and increasing membrane rigidity. This altered membrane stiffness compromises RBC deformability, potentially contributing to hemolytic anemia observed in patients (42, 43). The abnormal sterol incorporation disrupts membrane fluidity, which is critical for RBCs to traverse microvasculature efficiently (25). Such rigidity may also predispose RBCs to premature clearance, exacerbating anemia and related hematologic symptoms. The reduced deformability of RBCs caused by high levels of phytosterol leads to impaired microcirculation. RBCs with diminished flexibility face difficulty navigating narrow capillaries, resulting in microvascular obstruction and tissue hypoxia. This microcirculatory disturbance can contribute to clinical manifestations such as splenomegaly and arthralgia. Moreover, the impaired passage of rigid RBCs through the spleen may enhance their destruction, further aggravating anemia in sitosterolemia patients (44). Platelet abnormalities in sitosterolemia include defects in adhesion, aggregation, and secretion, which are crucial for normal hemostasis. Elevated phytosterol levels disrupt platelet membrane composition, impairing receptor function and signaling pathways necessary for platelet activation. Proteomic analyses indicate that thrombocytopenia in sitosterolemia is not due to intrinsic platelet defects but rather secondary to lipid-induced alterations affecting platelet function and lifespan. These dysfunctions may increase bleeding risk or contribute to thrombotic complications (45). Alterations in membrane lipid composition caused by cumulation of phytosterol affect membrane dynamics, promoting a prothrombotic state. The increased rigidity and altered membrane microdomains can enhance platelet activation and aggregation under pathological conditions, elevating thrombosis risk. Additionally, these membrane abnormalities may influence endothelial interactions and coagulation cascades, further predisposing patients to premature atherosclerosis and thrombotic events commonly seen in sitosterolemia.

In conclusion, the pathological role of abnormal phytosterol accumulation in sitosterolemia is multifaceted, involving intricate molecular and cellular mechanisms that collectively drive disease progression. Phytosterols disrupt membrane biophysics by embedding into lipid bilayers, increasing membrane rigidity and reducing fluidity. Such alterations impair membrane protein functions and signal transduction, triggering endoplasmic reticulum stress, oxidative stress, and activation of inflammatory pathways including TLR and NLRP3 inflammasomes. These events promote macrophage activation and foam cell formation, ultimately contributing to endothelial dysfunction and atherosclerosis. Importantly, the interplay between abnormally elevated levels of phytosterol and mTOR signaling emerges as a critical nexus in disease pathogenesis. Aberrant mTORC1 activation suppresses autophagy, exacerbating lipid metabolic disturbances and lysosomal dysfunction, which amplifies cellular stress and inflammation in a vicious cycle. Moreover, the hematological abnormalities induced by phytosterols—such as increased erythrocyte membrane rigidity impairing microcirculation and altered platelet membrane fluidity enhancing thrombogenicity—synergize with endothelial dysfunction to elevate cardiovascular risk. This convergence of vascular and hematologic dysfunction highlights the systemic nature of sitosterolemia and the necessity of comprehensive therapeutic strategies. The pathophysiological consequences in sitosterolemia are presented in Figure 1C.

## 3 Clinical features of sitosterolemia

### 3.1 Typical clinical presentation spectrum: from childhood to adulthood

Sitosterolemia displays a remarkably diverse phenotypic landscape (Table 1), with clinical onset spanning from early pediatric years to mature adulthood. Although symptoms frequently emerge prior to puberty, definitive diagnosis is often postponed or overlooked, leading to a high rate of initial misclassification as FH (5). The age at which the condition is identified varies extensively, ranging from infancy to the seventh decade of life, with reported cohorts indicating a median diagnosis between 8 and 13 years (6, 46). Characteristic findings encompass tendon and tuberous xanthomas that can develop during childhood, alongside hematologic disturbances such as macrothrombocytopenia and hemolytic anemia; notably, these blood disorders may manifest even without evident hypercholesterolemia (47, 48). A substantial fraction of children and adult cases present with either clinical or subclinical ASCVD, valvular heart disease and atherosclerosis, underscoring the significant threat of premature cardiac events (1). Clinical presentation remains highly heterogeneous: while some individuals are entirely asymptomatic carriers detected solely via cascade screening, others experience severe multi-system pathology involving complications such as arthralgia/arthritis, splenomegaly and stunted growth (2, 49, 50).

**Table 1:** Clinical manifestations of sitosterolemia.

Typical manifestations	Hematologic manifestations	Cardiovascular manifestations	Other manifestations
Xanthomas	Macrothrombocytopenia, Coombs-negative hemolytic anemia, stomatocytosis, splenomegaly, coagulation abnormalities	Valvular heart disease, atherosclerosis, atherosclerotic cardiovascular disease	Arthralgia, arthritis, splenomegaly, stunted growth

### 3.2 Hematologic manifestations and diagnostic pitfalls

Recent clinical observations have expanded the phenotypic spectrum to include significant hematological abnormalities, which often represent the initial clinical manifestations and contribute to frequent misdiagnosis (Table 2) (14, 49). Hematologic features in sitosterolemia patients include thrombocytopenia, Coombs-negative hemolytic anemia, macrothrombocytopenia, and stomatocytosis. These abnormalities can mimic other hematologic diseases such as immune thrombocytopenia (ITP), bone marrow failure syndromes like myelodysplastic syndrome (MDS), or inherited platelet disorders including Bernard–Soulier syndrome. For example, macrothrombocytopenia and bleeding diathesis have led to initial misdiagnoses of Bernard–Soulier variant syndrome, as illustrated in a case where genetic analysis ultimately revealed ABCG8 mutations confirming sitosterolemia (51). Similarly, chronic thrombocytopenia in familial clusters without ASCVD has been attributed to sitosterolemia upon genetic testing, underscoring the importance of considering this diagnosis in unexplained hematologic abnormalities (52).

**Table 2:** Hematologic manifestations and diagnostic pitfalls.

Hematologic manifestations	Features	Diagnostic pitfalls
Macrothrombocytopenia	Examination of peripheral blood smears indicates a decrease in platelet counts and the existence of abnormally large platelets. Treatment with ezetimibe can enhance platelet counts and normalize platelet size through the reduction of plasma plant sterol levels.	Immune thrombocytopenia, myelodysplastic syndrome, inherited platelet disorders (e.g., Bernard–Soulier syndrome)
Hemolytic anemia Stomatocytes	Coombs-negative hemolytic anemia. Peripheral blood smears are capable of revealing abnormal erythrocyte morphologies, specifically stomatocytes. The implementation of effective management strategies involving ezetimibe and dietary sterol restriction can reverse hemolytic anemia and enhance red cell morphology.	Hemolytic anemia
Splenomegaly	Effective management with ezetimibe and dietary sterol restriction has demonstrated reversal of splenomegaly.	Primary hematologic disorders
Coagulation abnormalities	The presence of macrothrombocytopenia and platelet dysfunction gives rise to bleeding diathesis. Conversely, elevated plasma sterols and the associated vascular changes render individuals prone to premature atherosclerosis and thrombosis. Ezetimibe therapy has been demonstrated to be effective in mitigating bleeding symptoms by augmenting platelet counts and function, and concurrently decelerating the progression of atherosclerosis via lipid reduction.	

The diagnostic challenge is compounded by the fact that many clinicians have limited awareness of sitosterolemia and the role of plant sterol measurement in evaluation. Conventional lipid profiles may not always reveal marked hypercholesterolemia, and xanthomas or cardiovascular manifestations may be absent or delayed, especially in pediatric patients or milder phenotypes (47, 53). Consequently, patients are often subjected to inappropriate treatments such as steroids, splenectomy, or statins without clinical improvement, further delaying correct diagnosis and targeted therapy (47). Peripheral blood smear examination can provide valuable clues, revealing stomatocytes and giant platelets, which should prompt consideration of sitosterolemia and subsequent genetic and biochemical testing.

#### 3.2.1 Platelet abnormalities: thrombocytopenia and macrothrombocytopenia

Platelet abnormalities in sitosterolemia frequently manifest as thrombocytopenia and macrothrombocytopenia, characterized by reduced platelet counts and the presence of abnormally giant platelets (45). These hematologic features are linked to mutations in *ABCG5*/*ABCG8* gene, which disrupt sterol metabolism and lead to lipid accumulation affecting platelet morphology and function. Macrothrombocytopenia may be misdiagnosed as ITP or Bernard–Soulier syndrome due to overlapping clinical presentations. Notably, treatment with ezetimibe has been shown to improve platelet counts and normalize platelet size by reducing plasma plant sterol levels, highlighting its therapeutic benefit in correcting platelet abnormalities (49, 52, 54). These findings underscore the importance of recognizing platelet size abnormalities in routine blood counts to prompt genetic testing.

#### 3.2.2 Red blood cell abnormalities: Coombs-negative hemolytic anemia and stomatocytosis

Sitosterolemia is associated with Coombs-negative hemolytic anemia often accompanied by morphological changes in erythrocytes such as stomatocytosis. The accumulation of phytosterols in red cell membranes disrupts membrane integrity, leading to premature red cell destruction and anemia. Cases have reported hemolytic anemia as a presenting feature, sometimes preceding or occurring without classical lipid abnormalities or xanthomas. Peripheral blood smears revealing abnormal erythrocyte shapes and giant platelets can provide early diagnostic clues. Effective management with ezetimibe and dietary sterol restriction has demonstrated reversal of hemolytic anemia and improvement in red cell morphology, emphasizing the need for early recognition and treatment to prevent complications (47, 55, 56).

#### 3.2.3 Splenomegaly

Splenomegaly in sterolemia is also among the manifestations of hematological abnormalities. Some newly-diagnosed patients may be misdiagnosed with primary hematologic disorders because of significant splenomegaly, and the diagnosis of sterolemia is subsequently confirmed via lipid profiling and genetic sequencing. These hematological findings demonstrate a favorable response to etafenib therapy, with platelet counts showing a marked recovery after treatment and splenomegaly being resolved. In clinical practice, for patients presenting with unexplained splenomegaly accompanied by hemolytic anemia or thrombocytopenia, when routine hematologic investigations fail to identify a clear etiology, sterolemia should be included in the differential diagnostic considerations.

#### 3.2.4 Coagulation abnormalities: concurrent bleeding and thrombotic risks

Sitosterolemia presents a paradoxical coagulation profile where patients may exhibit both bleeding tendencies and increased thrombotic risk. The presence of macrothrombocytopenia and platelet dysfunction contributes to bleeding diathesis, as observed in cases initially misdiagnosed with bleeding disorders like Bernard–Soulier syndrome. Conversely, elevated plasma sterols and associated vascular changes predispose to premature atherosclerosis and thrombosis. The dual risk necessitates careful clinical evaluation and management to balance hemorrhagic and thrombotic complications. Ezetimibe therapy has been effective in mitigating bleeding symptoms by improving platelet counts and function, while also reducing atherosclerotic progression through lipid lowering. Understanding the interplay between sterol-induced platelet abnormalities and coagulation pathways remains critical for optimizing patient outcomes (14, 25, 51).

## 4 Strategies to improve diagnostic accuracy

### 4.1 Clinical warning signs: xanthomas, family history, and hematologic abnormalities

Clinical recognition of sitosterolemia often hinges on identifying key warning signs such as xanthomas, a positive family history, and refractory thrombocytopenia. Patients may present with cutaneous and tendon xanthomas, which are hallmark features but can be mistaken for FH (57). Hematologic abnormalities including macrothrombocytopenia and Coombs-negative hemolytic anemia are frequently observed and may be the initial manifestation, sometimes leading to misdiagnosis as ITP or Bernard–Soulier syndrome. Family history may be absent or unremarkable, complicating early detection. Therefore, clinicians should maintain a high index of suspicion for sitosterolemia in patients with unexplained hematologic abnormalities and xanthomas, especially when lipid profiles are atypical or unresponsive to standard therapies (49). Early identification of these clinical clues is crucial to prompt further biochemical and genetic evaluation.

### 4.2 Characteristic biochemical markers: elevated phytosterols levels

The definitive biochemical signature of sitosterolemia is the pathological cumulation of phytosterols (phytosterols) within plasma and peripheral tissues (3). Analytical assessment via gas chromatography-mass spectrometry (GC–MS) or liquid chromatography-mass spectrometry (LC–MS) characteristically demonstrates significantly elevated concentrations of phytosterols (sitosterol, campesterol, and related phytosterols) (6, 13, 50). Although hypercholesterolemia frequently co-occurs, the distinguishing diagnostic criterion remains the disproportionate rise in phytosterols levels. Standard lipid panels do not measure phytosterols, which are elevated in sitosterolemia. This limitation contributes to frequent misdiagnosis as FH or other dyslipidemias. Advanced methods such as GC–MS or LC–MS are required to quantify plasma phytosterols accurately, enabling definitive diagnosis and monitoring of treatment efficacy (58). GC–MS or LC–MS allows precise measurement of plasma levels of sitosterol, campesterol, and other phytosterols, which are markedly elevated in affected individuals. Elevated phytosterols levels above 10 μg/mL (1 mg/dL) are diagnostic and differentiate sitosterolemia from other lipid disorders such as FH (14). Research proposes specific serum sitosterol thresholds (e.g., ≥10 μg/mL) to differentiate genetically confirmed patients from carriers or healthy controls, noting that affected individuals often exhibit concentrations far exceeding these limits (8). Serial monitoring of sterol levels also serves to evaluate treatment efficacy, particularly in response to dietary modifications and ezetimibe therapy (53). Consequently, this biochemical profile not only facilitates diagnosis in cases presenting with clinical features overlapping other dyslipidemias but also serves as a critical metric for evaluating therapeutic response (6). Despite its diagnostic value, phytosterols testing is not routinely performed in many clinical settings, contributing to underdiagnosis. Incorporating plasma phytosterols analysis into the diagnostic workflow for patients with hypercholesterolemia or unexplained hematologic abnormalities can significantly improve detection rates.

### 4.3 Genetic diagnosis: *ABCG5*/*ABCG8* mutation

Genetic testing targeting the *ABCG5* and *ABCG8* genes is essential for definitive diagnosis and understanding the molecular basis of sitosterolemia. Whole-exome sequencing and targeted gene panels have identified numerous pathogenic variants, including novel mutations, expanding the genotypic spectrum of the disease. Genetic analysis not only confirms the diagnosis but also distinguishes sitosterolemia from phenotypically similar disorders like FH, guiding appropriate management (2). The identification of homozygous or compound heterozygous variants correlates with more severe biochemical and clinical phenotypes, including elevated phytosterols and hematologic abnormalities (52). Population studies have revealed enrichment of pathogenic *ABCG5*/*ABCG8* variants in certain ethnic groups, emphasizing the need for ancestry-informed genetic screening programs (59). Integration of genetic diagnostics into clinical practice facilitates early detection, family cascade screening, and personalized treatment strategies.

### 4.4 Diagnostic criteria and challenges in differential diagnosis

**Figure 3:**
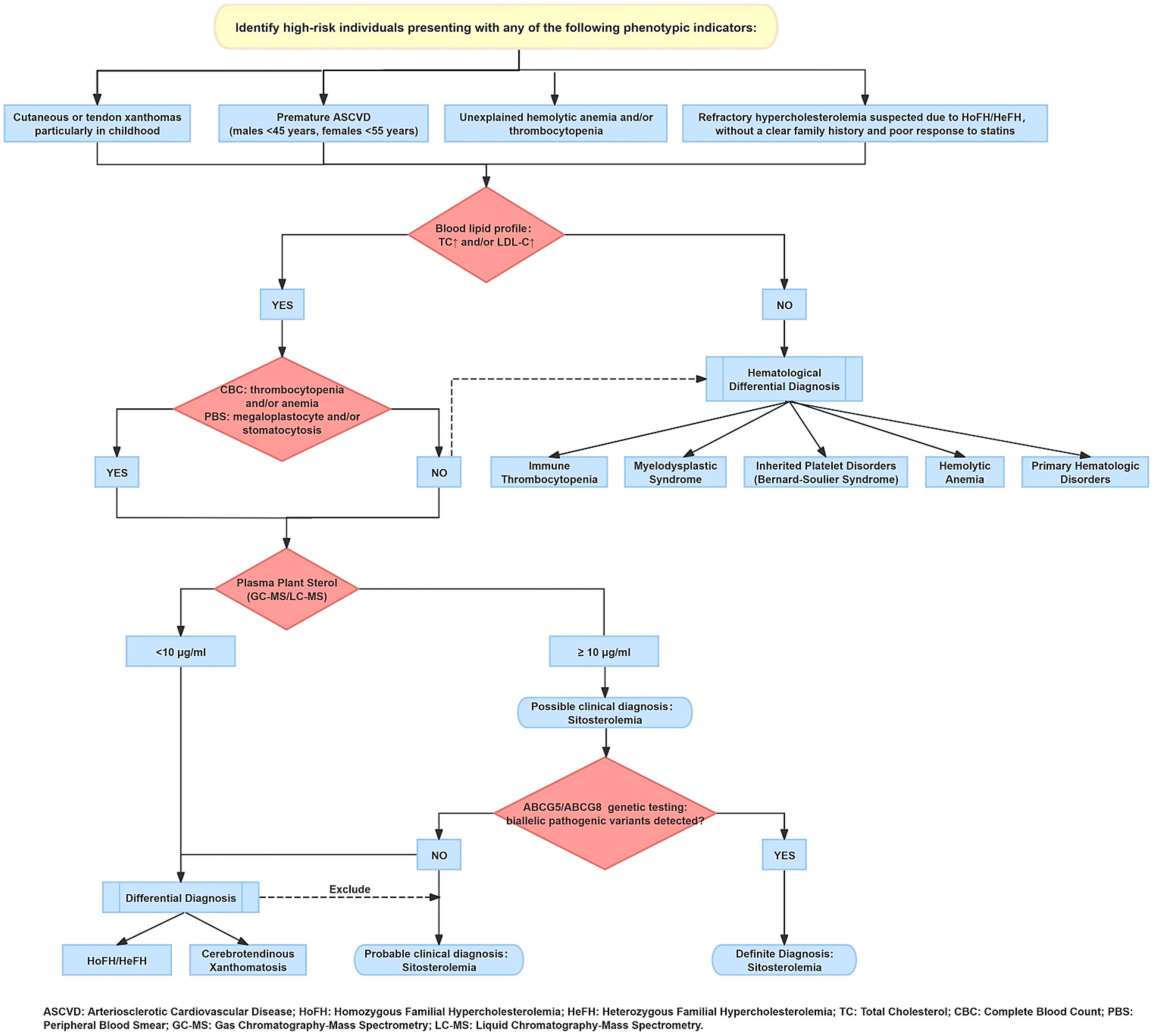
Diagnostic algorithm for suspected sitosterolemia.

Diagnosing sitosterolemia remains a formidable clinical challenge, primarily owing to its phenotypic mimicry of more prevalent disorders, notably FH (2). Consequently, numerous individuals endure misdiagnosis as FH cases for years or even decades, resulting in suboptimal management with statins that frequently yield limited therapeutic benefit (60). Definitive identification necessitates a triangulated approach: maintaining high clinical suspicion, biochemically verifying elevated circulating phytosterols, and employing molecular genetic assays to detect biallelic pathogenic mutations within the *ABCG5* or *ABCG8* loci (14). The differential diagnosis must encompass other etiologies presenting with xanthomas and hypercholesterolemia, such as cerebrotendinous xanthomatosis, alongside distinct causes of hemolytic anemia and thrombocytopenia (48). Integrating A*BCG5/ABCG8* sequencing into the diagnostic algorithm for suspected monogenic dyslipidemias is paramount, particularly for patients exhibiting atypical responses to conventional lipid-lowering regimens or concurrent hematologic anomalies (1, 61). Such diagnostic precision is indispensable for implementing targeted interventions, specifically ezetimibe administration coupled with a diet restricted in phytosterols, which constitute the foundational pillars of effective disease management (62). Diagnostic algorithm for suspected sitosterolemia is presented in Figure 3.

## 5 *ABCG5*/*ABCG8* gene mutation spectrum

### 5.1 Overview of mutation types: missense, nonsense, frameshift, and splice-site mutations

The mutational landscape of sitosterolemia exhibits considerable heterogeneity, involving a broad array of pathogenic alterations within the *ABCG5* and *ABCG8* genes (Figure 2, Supplementary Table 3). These genetic defects encompass nonsense, missense, frameshift, splice-site variants and large deletions, each compromising the functional integrity of the sterolin-1/sterolin-2 heterodimer essential for sterol efflux. Analysis of 155 cases from 133 families revealed 53 variants in *ABCG5* and 52 in *ABCG8*, with 33 and 29 protein-truncating variants respectively, indicating a high prevalence of loss-of-function mutations (63). The distribution of mutations shows ethnic variability; for example, c.1336C > T (p.R446*), c.1166G > A (p.R389H) and c.904 + 1G > A (p.IVS7 + 1G/A) in *ABCG5* are common in East Asians, whereas c.1083G > A (p.W361*), c.320C > G (p.S107*) and c.1715 T > C (p.L572P) in *ABCG8* predominate in Europeans (63). This broad mutation spectrum underscores the genetic heterogeneity of sitosterolemia and the importance of comprehensive genetic screening for diagnosis.

#### 5.1.1 Hotspot regions and functional domain localization of missense mutations

Missense mutations in *ABCG5* and *ABCG8* tend to cluster in critical functional domains essential for transporter activity. These hotspots often affect the ATP-binding cassette and transmembrane domains responsible for heterodimer formation and sterol transport. Structural and functional analyses suggest that such missense variants disrupt the heterodimerization of *ABCG5*/*ABCG8* impairing sterol efflux and leading to sterol cumulation (3). For instance, mutations like p.R419H in *ABCG5* have been linked to altered protein conformation and function, contributing to the sitosterolemia phenotype (64). Identifying these hotspots facilitates understanding of genotype–phenotype correlations and guides functional studies.

#### 5.1.2 Pathogenic mechanisms of nonsense mutations and premature stop codons

Nonsense mutations introducing premature termination codons (PTCs) in *ABCG5* or *ABCG8* lead to truncated, nonfunctional proteins or trigger nonsense-mediated mRNA decay, resulting in loss of transporter function. Such loss-of-function mutations severely impair the heterodimeric *ABCG5*/*ABCG8*, transporter responsible for sterol excretion, causing phytosterol accumulation (65). Research has documented nonsense mutations such as c.1234C > T (p.R412*) in *ABCG8* and c.1336C > T (p.R446*) in *ABCG5*, alongside missense changes like c.1337G > A (p.R446Q) in *ABCG5* (13, 48, 63). For example, the novel homozygous nonsense mutation c.1769C > A (p.S590*) in *ABCG5* was shown to hinder heterodimer function and sterol transport activity (65). These mutations are frequently observed in sitosterolemia and are critical for establishing molecular diagnosis and understanding disease severity.

#### 5.1.3 Molecular consequences of frameshift mutations and splice site variations

Frameshift mutations and splice site variants in *ABCG5*/*ABCG8* disrupt the reading frame or normal mRNA splicing, leading to aberrant or truncated proteins. Splice site mutations such as c.965-1G > A and c.323-1G > C in *ABCG8* have been reported to cause exon skipping or intron retention, resulting in defective protein products (66). Frameshift mutations similarly produce truncated proteins lacking essential domains, impairing transporter function. These molecular alterations compromise sterol efflux, contributing to sitosterolemia pathogenesis. Functional assays and bioinformatics analyses are essential to characterize these variants and their impact on protein structure and function.

#### 5.1.4 Rare reports of large fragment deletions and copy number variations (CNVs)

Large deletions and CNVs in *ABCG5* and *ABCG8* are rare but have been identified as pathogenic mechanisms in sitosterolemia. For instance, a compound heterozygous case involving an *ABCG5* exons 1–4 deletion alongside a nonsense mutation demonstrated markedly elevated plasma phytosterol levels and severe clinical manifestations (67). However, such large-scale genomic alterations are infrequently reported compared to point mutations. Their detection requires specialized techniques like multiplex ligation-dependent probe amplification or whole-genome sequencing. Recognizing CNVs expands the mutational landscape and improves genetic diagnosis accuracy in sitosterolemia.

### 5.2 Special mutation combinations and compound heterozygosity patterns

#### 5.2.1 Homozygous mutations and compound heterozygous mutations: clinical differences

Homozygous mutations in *ABCG5* or *ABCG8* typically result in classical sitosterolemia with severe phenotypes such as tendon xanthomas, hypercholesterolemia, and hematologic abnormalities including thrombocytopenia and hemolytic anemia (66). Compound heterozygous mutations, involving two different pathogenic variants on each allele, also cause sitosterolemia but can present with variable clinical severity and phenotypes. For example, patients with compound heterozygous *ABCG5* mutations showed diverse clinical manifestations, including xanthomas and hematologic symptoms, with some mutations causing structural instability of the sterolin proteins, affecting transporter function (13). Notably, clinical differences between homozygous and compound heterozygous patients are often subtle, and both groups can exhibit elevated plasma phytosterols and cardiovascular complications. However, the genotype–phenotype correlation remains inconsistent, suggesting that other genetic or environmental factors may modulate disease expression (68).

#### 5.2.2 Rare cases of dual-gene (*ABCG5*/*ABCG8*) mutations

Although sitosterolemia is caused by mutations in either *ABCG5* or *ABCG8*, rare cases with mutations in both genes have been reported, complicating the genetic landscape of the disease. A notable case described compound heterozygous mutations in *ABCG5*, with one variant inherited from each parent, alongside variants in *ABCG8*, highlighting the possibility of digenic inheritance (6, 9, 13). Such dual-gene mutations may disrupt the formation of the *ABCG5*/*ABCG8* heterodimer more severely, leading to impaired sterol transport and more pronounced clinical features. This discovery challenges traditional concepts of disease inheritance, suggesting that in certain cases, a heterozygous mutation in either gene may lead to functional loss through synergistic effects, thereby triggering the disease. These findings significantly expand our understanding of the genetic etiology of glucosterolemia. These rare cases emphasize the need for comprehensive genetic screening of both genes in suspected sitosterolemia patients to ensure accurate diagnosis and personalized treatment strategies (66).

#### 5.2.3 Impact of mutation combinations on protein heterodimer assembly

*ABCG5* and *ABCG8* proteins form a functional heterodimer essential for the excretion of sterols. Mutations affecting either subunit can impair heterodimer assembly, stability, and function. Loss of heterodimer integrity results in defective biliary and intestinal sterol excretion, causing excessively elevated levels of phytosterols in plasma and tissues. Structural modeling studies reveal that certain missense mutations induce conformational changes and reduce hydrogen bonding within the heterodimer, destabilizing the complex and hindering sterol transport (13). Compound heterozygous mutations often lead to unstable heterodimers, as seen with variants like p.M99R in *ABCG5*, which disrupt tertiary structure and phosphorylation sites critical for protein stability and function (4). The nonsense mutation c.1769C > A (p.S590*) in the *ABCG5* gene results in the production of a truncated protein that is unable to form a stable heterodimer with ABCG8, thereby losing its ability to transport sterols (65). Another missense mutation in the *ABCG5* gene, p.I68N, is also believed to potentially interfere indirectly with the normal assembly of heterodimers by affecting protein folding or stability (60). These findings highlight the diversity of mutations affecting protein structure and underscore the critical role of heterodimer assembly integrity in maintaining sterol transport function. Understanding the molecular consequences of specific mutation combinations is crucial for elucidating disease mechanisms and developing targeted therapies.

#### 5.2.4 Synergistic effects of loss-of-function mutations

The concept of “synergistic effects” discussed in the literature primarily focuses on interactions between functional loss mutations. For instance, in compound heterozygous mutations, two distinct functional loss mutations (e.g., a missense mutation and a nonsense mutation) may collectively lead to complete loss of protein function through different molecular mechanisms. Some variants may alter protein function in a way that partially compensates or exacerbates the defect. For instance, compound heterozygous mutations can include one loss-of-function and one variant of uncertain significance or mild effect, resulting in intermediate phenotypes with variable clinical severity. A typical example is the compound heterozygous mutations c.1337G > A (p.R446Q) and c.1396G > C (p.A466P) in the *ABCG5* gene; structural modeling predicts that both missense mutations induce conformational changes and reduced hydrogen bonding, thereby collectively compromising protein stability and producing a synergistic pathogenic effect (13). Furthermore, frameshift events contribute to transporter dysfunction; notably, a complex case revealed a novel deletion in *LDLR* occurring concurrently with a pathogenic *ABCG8* splice-site variant (69). This synergistic effect influences sterol metabolism, clinical manifestations, and response to treatments like ezetimibe. Additionally, although gain-of-function mutations are uncommon in sterolemia, polymorphisms in the *ABCG5*/*ABCG*8 gene (non-pathogenic variants) are associated with plasma cholesterol levels and gallstone risk. These polymorphisms may modulate transporter activity and interact complexly with functional loss mutations, influencing the final disease phenotype (10). Thus, current understanding of “synergistic effects” primarily centers on how different functional loss mutations contribute to severe transporter dysfunction through additive or synergistic interactions. Further functional studies are needed to clarify how different mutation types interact and impact *ABCG5*/*ABCG8* transporter activity and patient outcomes.

### 5.3 Classification of variant types and pathogenicity assessment

The pathogenicity determination of variants associated with hyperglucosterolemia must strictly comply with the sequence variant interpretation guidelines jointly issued by the American College of Medical Genetics and Genomics (ACMG) and the American Society for Molecular Pathology (AMP). These guidelines categorize variants into five classes: Pathogenic, Likely Pathogenic, Variant of Uncertain Significance (VUS), Likely Benign, and Benign, through comprehensive evaluations of multiple evidence levels, including population frequency data (e.g., gnomAD database), computational prediction tools (e.g., PolyPhen-2, SIFT, MutationTaster), functional study evidence, familial co-segregation analysis, and allele-specific expression data.

Regarding the *ABCG5*/*ABCG8* genes, considering that hyperglucosterolemia is an autosomal recessive disorder, the pathogenicity assessment of homozygous or compound heterozygous mutations necessitates special attention to whether the variant leads to complete loss of protein function (e.g., nonsense mutations, frameshift mutations, classic splicing site mutations) or significant impairment of sterol transport activity. Moreover, the ACMG guidelines stress that for rare genetic disorders, when assessing variant pathogenicity, precedence should be given to co-segregation evidence between variants and disease phenotypes in reported case cohorts, as well as validation results from *in vitro* functional assays (e.g., cellular sterol efflux experiments). It is notable that, given the high conservation of *ABCG5*/*ABCG8* genes in populations, missense mutations located in critical transmembrane domains or ATP-binding sites are more prone to be classified as pathogenic.

#### 5.3.1 Defining the characteristics and evidence of pathogenic mutations

Identifiable pathogenic mutations serve as the cornerstone for molecular diagnosis of sterolosis. Their common features include mutation types that cause protein truncation or complete loss (such as nonsense mutations, frameshift mutations, and classic splicing site mutations), as well as missense mutations whose functional experiments have demonstrated significant impairment of the sterol transport activity of *ABCG5*/*ABCG8* heterodimers. In the *ABCG5* gene, well-characterized pathogenic mutations such as c.1336C > T (p.R446*) and c.1750C > T (p.R584*) have been reported to induce protein truncation by introducing premature termination codons, resulting in complete loss of function. In the *ABCG8* gene, mutations like c.1169G > A (p.T390*) and c.1760–1761delAT (p.T587Cfs13) have been repeatedly documented and exhibit complete co-association with hyperplastic sterolosis in family studies. Additionally, certain missense mutations, such as *ABCG8* c.1895 T > C (p.L632P) and c.1979G > A (p.R660Q), have been confirmed through *in vitro* cell experiments to significantly reduce protein expression levels or impair sterol efflux function, thus being classified as pathogenic. These mutations typically occur in highly conserved transmembrane helical regions or nucleotide-binding domains (NBDs), where amino acid substitutions disrupt protein folding, dimerization, or ATP hydrolysis functions. It is noteworthy that pathogenic mutations often manifest as homozygous or compound heterozygous states in patients and are highly associated with early-onset and severe clinical manifestations (such as tendon xanthomas and atherosclerosis).

#### 5.3.2 Challenges in determining likely pathogenic mutations

Likely pathogenic mutations account for a significant proportion of the variant spectrum in sterolosis, and their identification faces multiple challenges. According to ACMG guidelines, a mutation is considered possibly pathogenic when it meets criteria for moderate pathogenicity (e.g., PM2: extremely low frequency in the control population; PM3: located in a hotspot region for recessive disease-causing mutations; PP1: co-segregated within affected families), but lacks definitive functional validation or sufficient case support. For *ABCG5*/*ABCG8* genes, many missense mutations (e.g., *ABCG5* c.604G > A [p.G202R], *ABCG8* c.1199G > A [p.R400H]) exhibit extremely low frequencies (<0.001%) across multiple databases and are situated in highly conserved regions; however, no in vitro functional assays have directly demonstrated their impact on sterol transport activity, leading to their classification as possibly pathogenic. The main challenges in determination arise from three aspects: first, sterolosis is a rare disorder with limited patient samples, making it difficult to obtain robust evidence through large-scale family-based co-segregation analyses; second, some missense mutations may only partially impair protein function (e.g., reducing transport efficiency rather than complete loss), while existing functional assays (e.g., radioactive labeled sterol excretion experiments) lack sufficient sensitivity and standardization; finally, splice site mutations (e.g., c.79 + 5G > A in the intronic region) may affect mRNA splicing but lack RNA-level validation, making their pathogenicity difficult to determine. Therefore, for potentially pathogenic mutations, clinical genetic counseling should involve a comprehensive assessment based on the patient’s phenotype (e.g., plant sterol levels, age of xanthoma onset) and recommend further functional studies.

#### 5.3.3 Reclassification strategies for variants of uncertain significance (VUS)

Variants of uncertain significance (VUS) frequently appear in genetic testing reports for glucocorticoidosis, and their reclassification is a critical step in precision diagnosis. Common VUS reclassification strategies include: frequency updates based on large-scale population databases (e.g., gnomAD v4.0), integrated analysis using computational prediction tools (e.g., REVEL, CADD), family-based co-segregation studies, and validation through in vitro functional assays. For the *ABCG5*/*ABCG8* genes, common VUS variants include missense mutations in non-conserved regions (e.g., *ABCG5* c.1024A > G [p.I342V]), synonymous mutations (e.g., *ABCG8* c.1234C > T(p.R412*)), and mutations in non-classical splicing regions. The first step in reclassification involves assessing the variant frequency in healthy control populations: if the frequency exceeds 0.01% in gnomAD, the variant is more likely to be benign; if it is zero, further analysis is required. The second step involves performing consistency analysis using multiple computational prediction tools: if all tools predict the variant as pathogenic, the likelihood of pathogenicity increases. The third step is family-based investigation: if the variant co-segregates with disease phenotypes in patients (e.g., when the patient carries a homozygous VUS while both parents are heterozygous carriers), this supports the pathogenicity hypothesis. Finally, functional assays represent the gold standard: for instance, by constructing expression plasmids encoding mutant *ABCG5*/*ABCG8* and transfecting them into HEK293 cells, their ability to export [PID: 3H]-steroid can be evaluated. If the transport activity of the mutants is significantly lower than that of the wild type (<50%), they may be classified as potentially pathogenic. Additionally, RNA sequencing can assess the impact of VUS sites in the splicing region on mRNA splicing, thereby providing direct evidence for reclassification.

#### 5.3.4 Criteria for excluding benign variants and potential benign variants

Benign variants and potentially benign variants must be systematically excluded in genetic testing for glucocorticoidemia to avoid false-positive diagnoses. According to the ACMG guidelines, the criteria for identifying benign variants include: a frequency>5% in healthy control populations (BA1 evidence); no association with the disease in large case–control studies (BS2 evidence); and functional validation demonstrating no impact on protein function (BS3 evidence). For the *ABCG5*/*ABCG8* genes, the benign classification of reported variants such as *ABCG5* c.1810C > G (p.L604V) and *ABCG8* c.1895 T > C (p.L632P) remains controversial. However, certain variants, such as *ABCG5* c.604G > A (p.G202R), exhibit a frequency as high as 0.2% in the GnomAD East Asian population and are predicted as benign by multiple computational tools (e.g., SIFT, PolyPhen-2), thus being classified as potentially benign. The core criterion for exclusion is population frequency data: a variant with a frequency exceeding 0.5% in databases such as GnomAD and without any reported pathogenicity is generally considered benign. Additionally, synonymous mutations [e.g., *ABCG8* c.1234C > T (p.R412*)] that do not affect splicing and are located in non-conserved regions are also classified as benign. For potentially benign variants, family pedigree data should be considered: if the variant carried by the patient is in a translocation relative to a known pathogenic mutation (i.e., located on different alleles) and the patient exhibits mild or no phenotype, a benign diagnosis can be supported. It is important to note that certain low-frequency variants (with frequencies of 0.1–0.5%) may be misclassified as potentially benign and require further confirmation through functional assays (e.g., protein expression analysis). In clinical practice, both benign and potentially benign variants are typically not included in pathogenicity reports; however, their non-pathogenic significance must be explained to patients during genetic counseling.

### 5.4 Population distribution and mutation hotspots

In East Asian populations, particularly patients in China and Japan, the mutation spectrum of glucosterolemia exhibits distinct characteristics, with mutations in the *ABCG5* gene being predominant. Clinical analyses reveal that mutations in *ABCG5* predominate in Chinese, Japanese and Korean patients, with c.1336C > T (p.R446*) being one of the most common variants, alongside others such as c.1166G > A (p.R389H), c.904 + 1G > A (p.IVS7 + 1G/A) and c.751C > A (p.Q251*) (6, 8, 46, 57, 63). This mutation’s prevalence suggests a founder effect or population-specific genetic drift, emphasizing the need for targeted genetic screening in East Asian cohorts. Functional consequences include elevated plasma plant sterol levels and typical clinical manifestations like xanthomas and hematological abnormalities. Early identification of this mutation allows for precise diagnosis and effective treatment strategies, such as dietary management and ezetimibe therapy, improving patient outcomes.

The mutational profiles of hyperlipidemia in the Middle Eastern populations demonstrate distinctive genetic traits, especially attributable to consanguineous marriages and specific demographic compositions, which result in a notable enrichment of certain pathogenic mutations. Large-scale genomic investigations carried out in the Qatari population indicated that the frequency of carriers of pathogenic or potentially pathogenic variants in *ABCG5*/*ABCG8* is substantially higher compared to other global populations. The combined carrier rate is approximately 1:102, and the disease burden is around 1:7509, significantly surpassing the estimates from the GnomAD database (65). This research identified seven pathogenic/potentially pathogenic variants (three in *ABCG5* and four in *ABCG8*) and detected four newly predicted loss-of-function variants in the Qatari population (59). A study involving Turkish patients also emphasized the genetic heterogeneity within this population, identifying three novel variants in the *ABCG5* gene (c.161G > A, c.1375C > T, IVS10 - 1G > T) and one new variant in *ABCG8* (c.1762G > C) (2, 49). Moreover, genome-wide association studies (GWAS) have identified multiple independent loci associated with plant sterol levels in the Qatari population, including rs145164937 and rs4299376 at the *ABCG*5/*ABCG*8 locus, further validating the significance of these loci in Middle Eastern populations (2).

In European and North American populations, a notable founder mutation has been identified in the *ABCG8* gene (2). The *ABCG8* gene mutation c.1083G > A (p.W361*), c.320C > G (p.S107*), c.1715 T > C (p.L572P) and c.1844C > T (p.S615L) were predominantly observed in cases of European descent (63). This missense mutation affects the sterol transporter function of *ABCG8*, contributing to sitosterolemia pathogenesis. The founder effect is supported by the mutation’s clustering in specific European subpopulations, indicating inheritance from a common ancestor. Although less frequent than mutations in *ABCG5* in East Asians, *ABCG8* variants like c.320C > G (p. S107*) significantly contribute to disease prevalence in Europeans (70). Furthermore, reviews have indicated that polymorphisms in the *ABCG*5/*ABCG*8 gene are associated with abnormalities in plasma cholesterol metabolism and represent a susceptibility gene for cholesterolithiasis in populations across Europe, Asia, and South America (10). These findings collectively suggest that specific mutations in the *ABCG8* gene may be highly prevalent in European populations due to founder effects, contrasting markedly with the mutation profiles observed in East Asian populations. This highlights the importance of accounting for population-specific mutation hotspots when conducting genetic screening across different ethnic backgrounds.

The mutation landscape of *ABCG5* and *ABCG8* in African and Latin American populations remains incompletely characterized, representing a significant gap in sitosterolemia research. Limited case reports and genetic studies from these regions have identified few pathogenic variants, suggesting underdiagnosis or genetic heterogeneity (71). Recent genome-wide association studies in Middle Eastern cohorts have begun to uncover novel loci influencing phytosterol levels, but similar comprehensive analyses in African and Latin American populations are lacking (72). This paucity of data impedes understanding of population-specific mutation hotspots and the development of tailored diagnostic and therapeutic approaches. Expanding genetic screening and research efforts in these underrepresented populations are essential to elucidate the full spectrum of ABCG5/ABCG8 mutations and improve global sitosterolemia management.

## 6 Genotype–phenotype correlation

### 6.1 Research on the correlation between genotype and clinical phenotype severity

Investigating the link between distinct genotypes and the severity of clinical phenotypes in sitosterolemia remains a pivotal research focus. Although biallelic loss-of-function mutations generally underpin the classic presentation, considerable variability in disease severity persists. For instance, individuals harboring homozygous or compound heterozygous alterations demonstrate markedly elevated plasma sitosterol concentrations (>10 μg/mL) relative to carriers or wild-type subjects (8). The type of mutation may influence presentation. The study noted that mean platelet count was significantly lower and sitosterol levels higher in patients with homozygous variants compared to those with heterozygous variants (49). Furthermore, while the specific affected gene may associate with particular traits, substantial phenotypic overlap exists. The extensive heterogeneity observed—spanning from isolated thrombocytopenia to profound hypercholesterolemia and premature atherosclerosis—implies that modifier genes and environmental determinants substantially influence clinical outcomes (1, 47).

### 6.2 Exploration of phenotypic differences between *ABCG5* and *ABCG8* mutations

While pathogenic variants in either *ABCG5* or *ABCG8* underlie sitosterolemia, emerging evidence points to subtle phenotypic distinctions. An analysis of Chinese cohorts revealed that individuals harboring *ABCG5* mutations exhibited hematological disturbances, specifically thrombocytopenia and anemia, although these findings warrant further confirmation (6). Conversely, a systematic review centered on ABCG8 defects estimated a 6.8% prevalence of hemolytic anemia, underscoring a distinct hematologic link (70). Nevertheless, substantial phenotypic overlap persists, given that defects in both genes share the cardinal biochemical feature of plant sterols accumulation. Definitive characterization of consistent genotypic-phenotypic correlations requires large-scale cohort investigations; current data suggest that disease severity likely reflects a continuum driven by the specific mutation’s effect on transporter efficacy, rather than the identity of the affected gene alone (2, 11).

Although biallelic mutations in ABCG5 or ABCG8 are the recognized cause of sitosterolemia, the pronounced phenotypic heterogeneity seen among carriers with identical pathogenic variants implies a sophisticated interaction between genetic susceptibility and environmental or regulatory elements. Environmental influences, shifts in lifestyle-related factors, epigenetic control encompassing DNA methylation and non-coding RNA-mediated regulation of ABCG5/ABCG8 expression, along with concurrent variations in other lipid metabolism-associated genes, underlie the diverse phenotypes observed among individuals harboring the same mutation. In addition to classical transcriptional activation via Liver X Receptors (LXRs), recent data underscore the involvement of epigenetic processes in shaping the expression of these sterol transporters (73). This epigenomic configuration makes the locus responsive to modulations driven by lifestyle. For example, studies in animal models reveal that high-fat diets trigger site-specific hypomethylation in the first intron of Abcg5, paralleling increased expression of both genes (74). Corroborating this, large-scale epigenome-wide association studies (EWAS) have pinpointed specific CpG sites within the ABCG5 gene body whose methylation status strongly associates with plasma LDL-cholesterol levels and coronary artery disease susceptibility (75). These observations indicate that individual differences in DNA methylation may subtly adjust sterol efflux efficiency, thus influencing disease manifestation. Conversely, the role of non-coding RNAs remains poorly understood. To date, no particular microRNAs or long non-coding RNAs have been conclusively confirmed as direct modulators of *ABCG5*/*ABCG8* in phytosterolemia, highlighting a critical knowledge gap regarding post-transcriptional regulation. Integrated multi-omics approaches combining whole-genome sequencing with methylome and transcriptome analyses are needed to clarify how co-occurring variants in other lipid metabolism genes and epigenetic alterations jointly shape the variable penetrance of sitosterolemia.

The existing genotype–phenotype association data present notable limitations, which are mainly manifested in the following crucial aspects: Firstly, the quantity of specific cases available for in-depth analysis is relatively restricted, rendering it arduous to underpin comprehensive and reliable statistical inferences. Secondly, the published research literature is plagued by substantial publication bias, with a preference for reporting cases featuring significant or abnormal phenotypes. Meanwhile, numerous cases with atypical clinical manifestations or incomplete information may be inadequately presented, thereby undermining the overall representativeness of the database. Thirdly, the detection methodologies for phytosterols, a critical biomarker, vary in terms of availability and standardization across different studies and clinical institutions, which impacts the reliability and comparability of test results. Fourthly, in many case records, hematological parameters such as complete blood counts and related morphological details often lack sufficient information, impeding systematic phenotypic analysis from a hematological perspective. Fifthly, the absence of unified classification criteria for clinical phenotypic variability poses challenges to the precise comparison and summarization of phenotypic profiles across different studies and cases. Future research should establish standardized testing protocols, collect larger-scale case data samples, standardize the recording of critical hematological parameters, and develop a universally recognized classification system for clinical variability. This will facilitate a clearer elucidation of genotype–phenotype associations in glucosterolemia and provide a solid research foundation for optimizing diagnostic pathways and enhancing the accuracy of patient identification.

## 7 Heterozygous carriers: biochemical phenotype versus clinical disease

### 7.1 Biochemical phenotype of single heterozygous mutation carriers: subclinical hyperphytosterolemia

Individuals harboring a single heterozygous pathogenic variant in either *ABCG5* or *ABCG*8, historically regarded as asymptomatic, frequently present with a specific biochemical signature marked by increased circulating phytosterols, a state defined as subclinical hyperphytosterolemia. Empirical data indicate that heterozygotes typically display serum sitosterol concentrations substantially exceeding those of non-carriers, yet remaining below the markedly elevated levels observed in biallelic (homozygous or compound heterozygous) subjects (8). Moreover, extensive population-based and clinical screening efforts have identified a higher-than-anticipated frequency of loss-of-function variants in *ABCG5/G8*, linking these heterozygous states to dysregulated lipid profiles and an elevated susceptibility to cardiovascular pathology (9). Such biochemical findings imply that even one loss-of-function allele can partially compromise sterolin transporter efficacy, resulting in detectable sterol retention. Collectively, these findings underscore a sophisticated gene-dose relationship modulated by genetic modifiers, suggesting that monoallelic mutations drive a continuum of sterol trafficking abnormalities extending beyond the canonical homozygous disease presentation (76). Recognizing these biochemical states—mildly elevated circulating phytosterols in carriers suggests a likely gene dosage mechanism (9). The complexity of genetic mutations results in varying degrees of abnormalities in phytosterols metabolism, leading to a continuum of clinical manifestations rather than the traditional view of sitosterolemia. This is still an emerging research field, and more large-scale prospective studies are needed to confirm the exact clinical relevance, so as to avoid misleading readers on this issue.

### 7.2 Epidemiological evidence linking single heterozygous mutations to increased cardiovascular disease risk

Recent epidemiological evidence indicates that individuals harboring a single heterozygous pathogenic allele in either *ABCG5* or *ABCG*8 may exhibit heightened susceptibility to ASCVD. Extensive population-based investigations have demonstrated that even simple heterozygotes display aberrant lipid profiles, a factor potentially driving increased cardiovascular risk (9). While such carriers typically lack the overt clinical manifestations of sitosterolemia, including prominent xanthomas, the chronic, low-grade accumulation of atherogenic phytosterols likely fosters a pro-atherogenic milieu. This hypothesis is reinforced by data showing that the frequency of *ABCG5/ABCG8* variants exceeds prior estimates, especially within cohorts diagnosed with hypercholesterolemia; this suggests their role in population-level cardiovascular risk has been substantially underestimated (9). Furthermore, the observation that heterozygous mutation carriers can present with sitosterol concentrations surpassing established diagnostic thresholds obscures the distinction between carrier status and frank disease, thereby necessitating a reassessment of cardiovascular risk stratification protocols for this group (8).

### 7.3 Potential pathogenic mechanisms: haploinsufficiency, dominant-negative effects, and modifier genes

The pathological pathways through which a solitary heterozygous variant precipitates disease are complex, encompassing mechanisms such as haploinsufficiency, dominant-negative interference, and modulation by secondary genetic factors. The detection of intermediate plant sterol concentrations in carriers corroborates the haploinsufficiency hypothesis, wherein a 50 % decrement in functional transporter abundance results in a measurable biochemical anomaly (9). Moreover, given that the *ABCG5/G8* complex operates as a heterodimer, specific missense alterations may theoretically induce a dominant-negative impact; this occurs when mutant subunits associate with wild-type products from the intact allele, consequently compromising the integrity of the entire assembly (4). Additionally, the substantial phenotypic variability noted even among individuals with biallelic mutations implies the involvement of both genetic and environmental modifiers. In heterozygous subjects, concurrent genetic variants influencing lipid homeostasis or elevated dietary phytosterol consumption could potentially amplify the phenotypic manifestation of a single defective allele, thereby shifting subtle biochemical deviations into the realm of clinical relevance (14).

### 7.4 Clinical implications: impact on screening, diagnosis, and family genetic counseling

The realization that solitary heterozygous mutations can elicit both biochemical alterations and potential clinical manifestations carries significant weight for screening protocols, diagnostic strategies, and genetic counseling practices. First, this evidence supports incorporating ABCG5 and ABCG8 into genetic panels for individuals presenting with hypercholesterolemia, particularly those exhibiting an inadequate response to statin therapy. Detecting a heterozygous variant in such cases may clarify the underlying phenotype and steer therapeutic decisions toward ezetimibe (14, 60). Second, diagnostic algorithms likely require revision. Although serum sitosterol concentrations exceeding 10 μg/mL were historically regarded as definitive for biallelic disease, such levels occasionally appear in carriers. This observation underscores the necessity of confirmatory genetic testing and suggests that screening cutoff values may need adjustment (8). Finally, within the context of familial genetic counseling, identifying a heterozygous variant in a proband mandates cascade screening among relatives. Counseling frameworks must now encompass the risk of subclinical hyperphytosterolemia and its associated cardiovascular implications for carriers, transcending the traditional binary classification of “affected” versus “unaffected” characteristic of classic recessive disorders (9).

## 8 Treatment and prognosis

### 8.1 Dietary intervention: importance of low plant sterol diet and challenges of dietary management

Dietary intervention remains the primary strategy of sitosterolemia treatment, emphasizing a low plant sterol diet to reduce sterol accumulation and associated complications. Individuals diagnosed with plant sterolemia should refrain from consuming foods abundant in phytosterols, such as corn oil, sesame seeds, peanuts, soybeans, rapeseed oil, sesame oil, rice oil, margarine, avocado, chocolate, nuts, cereal and shellfish. In contrast, other vegetables and fruits, like potatoes, carrots, and apples, contain relatively lower levels of phytosterols. Apart from phytosterols, they should also avoid foods with high cholesterol content, including animal liver and eggs. Clinical data show that restricting dietary intake of phytosterols significantly lowers plasma sterol levels and improves symptoms such as xanthomas and hematologic abnormalities (53). Dietary therapy alone can reduce total cholesterol and LDL-C by over 50%, underscoring its vital role in management (53).

However, adherence to such dietary restrictions can be challenging due to the ubiquitous presence of phytosterols in common foods, including fruits, vegetables, and vegetable oils. The core challenges in dietary guidance for children with stigesterolemia are as follows: (1) There is a significant dearth of information regarding food sterol content. Most common food databases only specify cholesterol levels, with scarcely any tables indicating the quantities of plant sterols (e.g., stigesterol, soy sterol, rapeseed sterol, etc.). This makes it arduous for parents to identify “high-sterol” foods, presenting substantial challenges in daily food selection. (2) Common “healthy diet” recommendations often conflict with the specific dietary requirements of this disease. For example, for typical children, it is generally recommended to increase the consumption of vegetable oils, nuts, legumes, whole grains, and dark-colored vegetables—precisely the foods that are high in plant sterols. Parents frequently engage in inappropriate feeding practices due to misunderstandings of the concept of “health.” (3) The hidden sources of high-plant sterol foods are extensive. Commercial baked goods, margarine, vegetable fat powders, and certain brands of milk beverages contain added plant sterol fortifiers; the use of vegetable oils for frying when dining out is also unavoidable. (4) Feeding guidelines vary considerably across different age groups, yet clinically, there is often a lack of specific protocols. For instance, infants and young children with sitosterolemia are recommended to consume special medical formulas that are either devoid of or contain extremely low levels of phytosterols (e.g., amino acid-based formulas or designated low-sterol formulas). During complementary feeding, legumes, nut purees, and whole grains should be avoided, while low-sterol fruits, vegetables, and animal-based foods should be prioritized. (5) Blood sterol monitoring lags behind dietary deviations. Elevated plasma plant sterol levels often only manifest after several weeks, and most affected children exhibit no obvious symptoms. Consequently, parents find it difficult to directly perceive the consequences of dietary errors, which reduces their motivation to adhere to dietary guidance.

Meanwhile, the difficulty lies in balancing nutritional adequacy with sterol restriction, necessitating multidisciplinary support. Children need to strike a balance between strictly limiting phytosterols intake and maintaining adequate nutrition, with individualized dietary plans that require regular reassessment. Animal-derived high-quality proteins should replace plant-based proteins. Main meals should consist primarily of refined grains (white rice and wheat products) to meet caloric needs, supplemented with small amounts of low-sterol whole grains; vegetables should mainly be light-colored varieties, while dark-colored vegetables should be blanched to reduce sterol content and consumed in moderation; Fruits should be consumed in adequate daily amounts but avoided when paired with nuts.

Moreover, long-term compliance of dietary intervention are critical for sustained benefits. Yet it challenging to effectively implement long-term dietary therapy for patients with sitosterolemia, especially in children. Children’s long-term compliance is influenced by factors such as family awareness levels, economic status, social support, cooperation between the child and family, psychological state, health education for patients, as well as treatment and follow-up practices. Therefore, when developing a dietary treatment plan, the medical team needs to conduct individualized assessment and intervention for different patients, provide targeted health education, and maintain regular follow-up to continuously supervise and adjust the plan, so as to improve patients’ long-term compliance.

### 8.2 Pharmacological treatment: ezetimibe and bile acid sequestrants

Contemporary pharmacological therapeutic strategies prioritize the attenuation of intestinal sterol uptake. Pharmacotherapy serves as the cornerstone in the management of hypercholesterolemia, predominantly encompassing ezetimibe and bile acid chelators. Ezetimibe stands as the current first-line treatment, operating through the inhibition of the Niemann-Pick C1-Like 1 (NPC1L1) transporter on enterocytes, which serves as the principal gateway for dietary cholesterol and plant sterol absorption (62). By obstructing this specific absorptive route, ezetimibe significantly lowers circulating concentrations of both cholesterol and phytosterols. This precise mechanism accounts for its superior efficacy in sitosterolemia relative to statins, which mainly suppress endogenous cholesterol synthesis without correcting the fundamental absorptive anomaly (60). Consequently, combining ezetimibe with a diet restricted in phytosterols constitutes the current standard of care. A standard daily dose of 5–10 mg notably reduces both serum cholesterol and phytosterol levels, while simultaneously ameliorating hematological abnormalities and atherosclerotic risk. Nevertheless, substantial individual variability is present, which may necessitate higher doses or combination therapy for certain patients. Ezetimibe has demonstrated significant reductions in plasma phytosterols and LDL cholesterol, with clinical improvements in hematologic parameters such as thrombocytopenia and anemia (52). The adverse reactions of ezetimibe is generally well-tolerated, although occasional headaches or diarrhea may transpire.

Bile acid sequestrants, such as cholestyramine, function by binding luminal bile acids to promote their fecal elimination; this process compels the liver to consume cholesterol for *de novo* bile acid production, potentially diminishing hepatic cholesterol reserves (6). Bile acid chelators are typically employed as second-line or adjunctive therapy (77). The daily dosage of cholestyramine is 4–15 grams for adults and 4–8 grams for children, commencing with a low-dose regimen of 4 grams per day and gradually increasing. Although the efficacy of these agents is well-documented, gastrointestinal adverse effects (e.g., abdominal distension, constipation) and drug interactions limit their long-term application. Regarding the other adverse reactions of bile acid chelators, attention should be given to the risk of fat-soluble vitamin deficiencies.

Treatment selection ought to be customized according to individual patient characteristics, such as patient tolerance, severity, and response, with ezetimibe generally preferred due to better efficacy and safety profiles. For patients who exhibit a poor response to ezetimibe monotherapy or have severe hypercholesterolemia, combination therapy with bile acid chelators can be considered (53). During treatment, regular monitoring of phytosterol levels, lipid profiles, and liver function is crucial for dosage adjustment and efficacy evaluation.

### 8.3 Reversal of hematologic abnormalities and long-term management

Hematologic manifestations such as macrothrombocytopenia and hemolytic anemia frequently accompany sitosterolemia and can be reversed with appropriate treatment. Studies report normalization of platelet counts and resolution of anemia following ezetimibe therapy combined with dietary restrictions (49). Long-term management requires continuous monitoring of hematologic parameters and lipid profiles to prevent relapse. Early diagnosis and intervention are crucial to avoid irreversible organ damage. Genetic counseling and family screening are also integral to long-term care, given the autosomal recessive inheritance pattern. Sustained treatment adherence is essential to maintain hematologic and cardiovascular health over time (52).

### 8.4 Treatment of other symptoms

Beyond lipid abnormalities and hematologic issues, sitosterolemia patients may present with xanthomas, arthralgia, and hepatosplenomegaly. Treatment of these symptoms involves addressing the underlying sterol accumulation through diet and pharmacotherapy, which often leads to regression of xanthomas and improvement in joint symptoms (55). Symptomatic management may include analgesics for arthralgia and monitoring for hepatic involvement. Early intervention can prevent progression to severe complications such as premature atherosclerosis and organ dysfunction. Multidisciplinary care is recommended to manage the diverse clinical manifestations effectively (56).

## 9 Future directions

### 9.1 Novel biomarkers: prospects of oxysterols and Lipidomics

Emerging research into novel biomarkers such as oxidized sterols (oxysterols) and comprehensive lipidomic profiling holds promise for enhancing diagnostic precision and understanding disease pathophysiology. Oxysterols, derivatives of cholesterol oxidation, may contribute to the atherogenic risk in sitosterolemia and serve as potential indicators of disease severity and progression (25). Lipidomics, the large-scale study of lipid species, can uncover distinct lipid signatures associated with ABCG5/ABCG8 dysfunction, providing insights into metabolic disturbances beyond traditional sterol measurements. Although these approaches are still in early investigative stages, they offer the potential to refine risk stratification, monitor therapeutic response, and identify novel therapeutic targets. Continued advancements in analytical technologies and validation in clinical cohorts are needed to translate these biomarkers into routine diagnostic tools.

### 9.2 Prospects for potential targeted therapies based on pathogenic mechanisms

Future investigations should prioritize identifying key molecular nodes where membrane alterations, inflammatory signaling, and mTOR pathway dysregulation intersect. Targeting these integrative hubs may enable the development of novel interventions aimed at restoring membrane fluidity, modulating inflammasome activity, and normalizing mTOR signaling. Such targeted therapies hold promise for improving clinical outcomes in sitosterolemia patients by mitigating both vascular and hematologic complications.

Future therapeutic paradigms are increasingly focused on directly rectifying the fundamental transporter malfunction. Recent elucidations of the structure–function dynamics within the *ABCG5/G8* heterodimer—particularly regarding pivotal regions such as the transmembrane polar relay required for ATPase function—have established a robust framework for the design of pharmacochaperones (12). These low-molecular-weight agents hold the potential to stabilize distinct mutant conformations, thereby facilitating correct protein folding, dimer assembly, and subsequent trafficking to the plasma membrane. Moreover, the widespread occurrence of individuals harboring single *ABCG5* or *ABCG8* variants, coupled with their link to dysregulated lipid metabolism, underscores the applicability of gene-specific interventions across a wider demographic (9). In instances of severe pathology, gene therapy aimed at introducing functional *ABCG5* or *ABCG8* copies offers a promising curative avenue, although this approach currently remains in preliminary investigative phases. Ultimately, tailoring treatment strategies to the precise molecular anomaly (e.g., distinguishing between nonsense and missense mutations) may significantly enhance future clinical management outcomes (11).

## 10 Conclusion

In summary, sitosterolemia is a well-defined monogenic condition resulting from biallelic pathogenic variants in either ABCG5 or ABCG8, with its primary pathophysiological defect lying in impaired plant sterol excretion. Comprehensive genetic analysis remains the diagnostic gold standard for confirmation and classification. Notably, recent insights into the phenotypic profiles of monoallelic carriers have substantially reshaped our understanding of this disorder. These findings challenge the traditional autosomal recessive paradigm by revealing that certain heterozygous individuals may present with marked biochemical derangements and associated clinical risks. Consequently, clinicians and genetic counselors must adopt a more refined and vigilant strategy when assessing probands and their relatives.

Going forward, it is essential to integrate established knowledge of homozygous and compound heterozygous disease with the emerging complexities of the heterozygous state. Priority research directions should focus on clarifying the exact molecular pathways through which single mutant alleles influence phenotype, possibly via gene–gene or gene–environment interactions. Large-scale, longitudinal cohort studies are critically needed to firmly establish whether heterozygous carriage confers increased risk for hard cardiovascular outcomes. Moreover, this evolving genetic insight must inform the development of more effective, personalized therapeutic approaches tailored to the distinct risk profiles of both homozygous and heterozygous patients. Ultimately, deepening our comprehension of the *ABCG5/ABCG8* mutational landscape and underlying disease mechanisms will not only advance the management of sitosterolemia but also offer a valuable framework for investigating broader aspects of sterol metabolism and their contribution to ASCVD pathogenesis.
