# Supplementary material for: Molecular genetic basis and clinical heterogeneity of sitosterolemia: focusing on the mutation spectrum and pathogenic mechanisms of ABCG5/ABCG8 genes
Source: Front Nutr. 2026 Jul 17;13:1857512. doi: 10.3389/fnut.2026.1857512 (PMC13423872; doi:10.3389/fnut.2026.1857512)
Supplement: Supplementary file 1 [file Table_1.DOCX]

Supplement Table 1. *ABCG5*/*ABCG8* mutation spectrum

| **Gene** | **Chromosome** | **Strand** | **Location** | **Exon/Intron number** | **Nucleotide change** | **Effect of protein** | **ACMG** | **Mutation type** |
| --- | --- | --- | --- | --- | --- | --- | --- | --- |
| *ABCG5* | Chr2 | - | 43813157 | Exon 13 | c.1915delA | p.I639X | Likely Pathogenic | Nonsense |
| *ABCG5* | Chr2 | - | 43813181 | Exon 13 | c.1890delT | p.F630Lfs*8 | Pathogenic | Frameshift |
| *ABCG5* | Chr2 | - | 43813303 | Exon 13 | c.1769C>A | p.S590X | Likely Pathogenic | Nonsense |
| *ABCG5* | Chr2 | - | 43813310 | Exon 13 | c.1763-1G>A |  | Pathogenic | Splicing |
| *ABCG5* | Chr2 | - | 43814476 | Exon 12 | c.1762+1G>A |  | Pathogenic | Splicing |
| *ABCG5* | Chr2 | - | 43814504 | Exon 12 | c.1735G>T | p.E579X | Pathogenic | Nonsense |
| *ABCG5* | Chr2 | - | 43814557 | Exon 12 | c.1681dup | p.I561fs | Pathogenic | Frameshift |
| *ABCG5* | Chr2 | - | 43814561 | Exon 12 | c.1673_1677delCTTTT | p.P558Qfs*14 | Likely Pathogenic | Frameshift |
| *ABCG5* | Chr2 | - | 43814582 | Exon 12 | c.1657C>T | p.Q553X | Pathogenic | Nonsense |
| *ABCG5* | Chr2 | - | 43814589 | Exon 12 | c.1650A>C | p.R550S | VUS | Missense |
| *ABCG5* | Chr2 | - | 43819910 | Intron 11 | c.1649+3insT |  | Pathogenic | Splicing |
| *ABCG5* | Chr2 | - | 43819943 | Exon 11 | c.1621G>A | p.G541R | VUS | Missense |
| *ABCG5* | Chr2 | - | 43819976 | Exon 11 | c.635-153_1588del |  | Likely pathogenic | Splicing |
| *ABCG5* | Chr2 | - | 43819991 | Exon 11 | c.1573C>T | p.Q525X | Pathogenic | Nonsense |
| *ABCG5* | Chr2 | - | 43820036 | Exon 11 | c.1528C<A | p.H510N | Likely Benign | Missense |
| *ABCG5* | Chr2 | - | 43820036 | Exon 11 | c.1528C>G | p.H510D | VUS | Missense |
| *ABCG5* | Chr2 | - | 43820035 | Exon 11 | c.1528dup | p.H510fs | Pathogenic | Frameshift |
| *ABCG5* | Chr2 | - | 43820036 | Exon 11 | c.1528del | p.H510fs | Pathogenic | Frameshift |
| *ABCG5* | Chr2 | - | 43820101 | Exon 11 | c.1464-1G>C |  | VUS | Splicing |
| *ABCG5* | Chr2 | - | 43820102 | Exon 11 | c.1464-2A>G |  | Likely pathogenic | Splicing |
| *ABCG5* | Chr2 | - | 43822825 | Exon 10 | c.1435del | p.T479fs | Pathogenic | Frameshift |
| *ABCG5* | Chr2 | - | 43822881 | Exon 10 | c.1378_1379insT | p.K460fs | Likely pathogenic | Frameshift |
| *ABCG5* | Chr2 | - | 43822885 | Exon 10 | c.1375C>T | p.Q459X | Pathogenic | Nonsense |
| *ABCG5* | Chr2 | - | 43822886 | Exon 10 | c.1374C>G | p.Y458X | Pathogenic | Nonsense |
| *ABCG5* | Chr2 | - | 43822923 | Exon 10 | c.1337G>A | p.R446Q | Likely Pathogenic | Missense |
| *ABCG5* | Chr2 | - | 43822924 | Exon 10 | c.1336C>T | p.R446X | Pathogenic | Nonsense |
| *ABCG5* | Chr2 | - | 43823897 | Intron 9 | c.1325-3_c.1325-2delCA |  | Pathogenic | Splicing |
| *ABCG5* | Chr2 | - | 43823898 | Exon 9 | c.1324_1324+15del |  | Likely pathogenic | Splicing |
| *ABCG5* | Chr2 | - | 43823911 | Exon 9 | c.1323_1324+2del |  | Likely pathogenic | Splicing |
| *ABCG5* | Chr2 | - | 43823926 | Exon 9 | c.1311C>G | p.N437K | Pathogenic | Missense |
| *ABCG5* | Chr2 | - | 43823945 | Exon 9 | c.1292C>T | p.P431L | VUS | Missense |
| *ABCG5* | Chr2 | - | 43823964 | Exon 9 | c.1273C>T | p.Q425X | Pathogenic | Nonsense |
| *ABCG5* | Chr2 | - | 43823981 | Exon 9 | c.1256G>C | p.R419P | VUS | Missense |
| *ABCG5* | Chr2 | - | 43823981 | Exon 9 | c.1256G>A | p.R419H | VUS | Missense |
| **Gene** | **Chromosome** | **Strand** | **Location** | **Exon/Intron number** | **Nucleotide change** | **Effect of protein** | **ACMG** | **Mutation type** |
| *ABCG5* | Chr2 | - | 43823980 | Exon 9 | c.1255C<T | p.R419C | VUS | Missense |
| *ABCG5* | Chr2 | - | 43824015 | Exon 9 | c.1222C>T | p.R408X | Pathogenic | Nonsense |
| *ABCG5* | Chr2 | - | 43824020 | Exon 9 | c.1217G>A | p.R406Q | VUS | Missense |
| *ABCG5* | Chr2 | - | 43824071 | Exon 9 | c.1166G>A | p.R389H | Pathogenic | Missense |
| *ABCG5* | Chr2 | - | 43824099 | Exon 9 | c.1138del | p.L379_V380insX | Pathogenic | Nonsense |
| *ABCG5* | Chr2 | - | 43824216 | Intron 8 | c.1118+3A>C |  | VUS | Splicing |
| *ABCG5* | Chr2 | - | 43824218 | Exon 8 | c.1118+1G>C |  | Likely pathogenic | Splicing |
| *ABCG5* | Chr2 | - | 43824229 | Exon 8 | c.1108_1118+2del |  | Likely Pathogenic | Splicing |
| *ABCG5* | Chr2 | - | 43824352 | Exon 8 | c.985T>C | p.Y329H | VUS | Missense |
| *ABCG5* | Chr2 | - | 43824350 | Exon 8 | c.987C>A | p.Y329X | Likely Pathogenic | Nonsense |
| *ABCG5* | Chr2 | - | 43824359 | Exon 8 | c.978del | p.E326fs | Pathogenic | Frameshift |
| *ABCG5* | Chr2 | - | 43824423 | Exon 8 | c.914C>G | p.T305R | Likely Pathogenic | Missense |
| *ABCG5* | Chr2 | - | 43824423 | Intron 7 | c.904+5G>C |  | VUS | Splicing |
| *ABCG5* | Chr2 | - | 43824887 | Intron 7 | c.904+2G>A |  | Pathogenic | Splicing |
| *ABCG5* | Chr2 | - | 43824888 | Exon 7 | c.904+1G>C |  | Pathogenic | Splicing |
| *ABCG5* | Chr2 | - | 43824888 | Exon 7 | c.904+1G>A |  | Pathogenic | Splicing |
| *ABCG5* | Chr2 | - | NA | Exon 7 | NA (288X (19bp insert)) |  | Pathogenic | Frameshift |
| *ABCG5* | Chr2 | - | 43824943 | Exon 7 | c.850G>A | p.G269R | VUS | Missense |
| *ABCG5* | Chr2 | - | 43825019 | Exon 7 | c.775-1G>A |  | Likely pathogenic | Splicing |
| *ABCG5* | Chr2 | - | 43826405 | Exon 6 | c.751C>T | p.Q251X | Pathogenic | Nonsense |
| *ABCG5* | Chr2 | - | 43826429 | Exon 6 | c.727C>T | p.R243X | Pathogenic | Nonsense |
| *ABCG5* | Chr2 | - | 43827982 | Exon 6 | c.634+1G>C |  | Likely pathogenic | Splicing |
| *ABCG5* | Chr2 | - | 43827982 | Exon 6 | c.634+1G>A |  | Likely pathogenic | Splicing |
| *ABCG5* | Chr2 | - | 43828041 | Exon 5 | c.575dup | p.I193fs | Likely pathogenic | Frameshift |
| *ABCG5* | Chr2 | - | 43828041 | Exon 5 | c.576del | p.I193fs | Likely pathogenic | Frameshift |
| *ABCG5* | Chr2 | - | 43828042 | Exon 5 | c.575del | p.Gly192fs | Pathogenic | Frameshift |
| *ABCG5* | Chr2 | - | 43828070 | Exon 5 | c.547C>T | p.R183X | Pathogenic | Nonsense |
| *ABCG5* | Chr2 | - | 43831768 | Exon 4 | c.501+1G>A |  | Likely pathogenic | Splicing |
| *ABCG5* | Chr2 | - | 43831834 | Exon 4 | c.436G>C | p.E146Q | VUS | Missense |
| *ABCG5* | Chr2 | - | 43831834 | Exon 4 | c.436G>T | p.E146X | Pathogenic | Nonsense |
| *ABCG5* | Chr2 | - | 43831869 | Exon 4 | c.403-2A>T |  | Likely pathogenic | Splicing |
| *ABCG5* | Chr2 | - | 43831982 | Exon 3 | c.367G>T (p.Glu123Ter) | p.E123X | Pathogenic | Nonsense |
| *ABCG5* | Chr2 | - | 43831994 | Exon 3 | c.351_354dup | p.Ala119fs | Likely pathogenic | Frameshift |
| *ABCG5* | Chr2 | - | 43822924 | Exon 3 | C336-337insA | C336-337insA | Pathogenic | Frameshift |
| *ABCG5* | Chr2 | - | 43832010 | Exon 3 | c.335dupA | p.V113fs | Pathogenic | Frameshift |
| *ABCG5* | Chr2 | - | 43832017 | Exon 3 | c.332G<A | p.G111E | Likely Pathogenic | Missense |
| **Gene** | **Chromosome** | **Strand** | **Location** | **Exon/Intron number** | **Nucleotide change** | **Effect of protein** | **ACMG** | **Mutation type** |
| *ABCG5* | Chr2 | - | 43832028 | Exon 3 | c.321del | p.T108fs | Pathogenic | Frameshift |
| *ABCG5* | Chr2 | - | 43832038 | Exon 3 | NA (exon 3 I/D) | exon 3 I/D | Pathogenic | Large deletion |
| *ABCG5* | Chr2 | - | 43832048 | Exon 3 | c.296T>G | p.M99R | VUS | Missense |
| *ABCG5* | Chr2 | - | 43832085 | Exon 3 | c.266-2A>G |  | Likely pathogenic | Splicing |
| *ABCG5* | Chr2 | - | 43837833 | Exon 2 | c.265+1G>A |  | Likely pathogenic | Splicing |
| *ABCG5* | Chr2 | - | 43837870 | Exon 2 | c.229G>T | p.E77X | Pathogenic | Nonsense |
| *ABCG5* | Chr2 | - | 43837908 | Exon 2 | c.191G>A | p.Trp64X | Likely pathogenic | Nonsense |
| *ABCG5* | Chr2 | - | 43837912 | Exon 2 | c.187C>T | p.Q63X | Pathogenic | Nonsense |
| *ABCG5* | Chr2 | - | 43837938 | Exon 2 | c.161G>A | p.W54X | Likely Pathogenic | Nonsense |
| *ABCG5* | Chr2 | - | 43837954 | Exon 2 | c.145del | p.H49fs | Pathogenic | Frameshift |
| *ABCG5* | Chr2 | - | 43837956 | Exon 2 | c.144-1G>A |  | Pathogenic | Splicing |
| *ABCG5* | Chr2 | - | 43838536 | Exon 1 | c.143+1G>T |  | Likely pathogenic | Splicing |
| *ABCG5* | Chr2 | - | 43838544 | Exon 1 | c.136del | p.S46fs | Pathogenic | Frameshift |
| *ABCG5* | Chr2 | - | 43838550 | Exon 1 | c.130T>C | p.S44P | VUS | Missense |
| *ABCG5* | Chr2 | - | 43838550 | Exon 1 | c.130T>G | p.S44A | VUS | Missense |
| *ABCG5* | Chr2 | - | 43838616 | Exon 1 | c.64C>T | p.Q22X | Pathogenic | Nonsense |
| *ABCG5* | Chr2 | - | 43838634 | Exon 1 | c.46C>T | p.Q16X | Pathogenic | Nonsense |
| *ABCG8* | Chr2 | + | 43844505 | Intron 1 | c.64-2A>G | IVS1-2A/G | Likely Pathogenic | Splicing |
| *ABCG8* | Chr2 | + | 43844563 | Exon 2 | c.120C>A | p.Y40X | Pathogenic | Nonsense |
| *ABCG8* | Chr2 | + | 43846214 | Exon 3 | c.225G>A | p.W75X | Likely Pathogenic | Nonsense |
| *ABCG8* | Chr2 | + | 43846238 | Exon 3 | c.250_280dup | p.K94delinsMX | Likely pathogenic | Nonsense |
| *ABCG8* | Chr2 | + | 43846246 | Exon 3 | g.12283-4insC | p.L86PfsX | Pathogenic | Frameshift |
| *ABCG8* | Chr2 | + | 43846309 | Exon 3 | c.320C>G | p.S107X | Pathogenic | Nonsense |
| *ABCG8* | Chr2 | + | 43851622 | Exon 4 | c.361C>T | p.R121X | Pathogenic | Nonsense |
| *ABCG8* | Chr2 | + | 43851643 | Exon 4 | c.382A>T | p.K128X | Likely pathogenic | Nonsense |
| *ABCG8* | Chr2 | + | 43851667 | Exon 4 | c.408del | p.Q137fs | Pathogenic | Frameshift |
| *ABCG8* | Chr2 | + | 43851751 | Exon 4 | c.490C>T | p.R164X | Pathogenic | Nonsense |
| *ABCG8* | Chr2 | + | 43851775 | Exon 4 | c.514C>T | p.Q172X | Likely Pathogenic | Nonsense |
| *ABCG8* | Chr2 | + | 43851806 | Exon 4 | c.547del | p.Q183Sfs*9 | Pathogenic | Frameshift |
| *ABCG8* | Chr2 | + | 43851812 | Exon 4 | c.551G>A | p.R184H | VUS | Missense |
| *ABCG8* | Chr2 | + | 43852353 | Exon 5 | c.562-1G>A |  | Likely pathogenic | Splicing |
| *ABCG8* | Chr2 | + | 43852376 | Exon 5 | c.584T>A | p.L195Q | Likely Pathogenic | Missense |
| *ABCG8* | Chr2 | + | 43852433 | Exon 5 | c.641del | p.S214fs | Likely pathogenic | Frameshift |
| *ABCG8* | Chr2 | + | 43852436 | Exon 5 | c.647_657dup | p.R220fs | Pathogenic | Frameshift |
| *ABCG8* | Chr2 | + | 43852475 | Exon 5 | c.683T>C | p.L228P | VUS | Missense |
| *ABCG8* | Chr2 | + | 43852483 | Exon 5 | c.691C>A | p.P231T | VUS | Missense |
| **Gene** | **Chromosome** | **Strand** | **Location** | **Exon/Intron number** | **Nucleotide change** | **Effect of protein** | **ACMG** | **Mutation type** |
| *ABCG8* | Chr2 | + | 43852491 | Intron 5 | c.694+5G>C | IVS5+5G/C | VUS | Splicing |
| *ABCG8* | Chr2 | + | 43852692 | Exon 6 | c.788G>A | p.R263Q | Likely Pathogenic | Missense |
| *ABCG8* | Chr2 | + | 43852715 | Exon 6 | c.811C>T | p.Q271X | Pathogenic | Nonsense |
| *ABCG8* | Chr2 | + | 43852785 | Exon 6 | c.881T>G | p.L294X | Likely pathogenic | Nonsense |
| *ABCG8* | Chr2 | + | 43852808 | Exon 6 | c.904C>T | p.Q302X | Pathogenic | Nonsense |
| *ABCG8* | Chr2 | + | 43871975 | Intron 6 | c.965-1G>C | IVS6-1G/C | Pathogenic | Splicing |
| *ABCG8* | Chr2 | + | 43871975 | Intron 6 | c.965-1G>A | IVS6-1G/A | Pathogenic | Splicing |
| *ABCG8* | Chr2 | + | 43872094 | Exon 7 | c.1083G>A | p.W361X | Pathogenic | Nonsense |
| *ABCG8* | Chr2 | + | 43873786 | Intron 8 | c.1212-1G>A | IVS8-1G/A | Likely Pathogenic | Splicing |
| *ABCG8* | Chr2 | + | 43873789 | Exon 9 | c.1214G>A | p.R405H | Likely Pathogenic | Missense |
| *ABCG8* | Chr2 | + | 43873800 | Exon 9 | c.1225A>G | p.N409D | VUS | Missense |
| *ABCG8* | Chr2 | + | 43873809 | Exon 9 | c.1234C>T | p.R412X | Pathogenic | Nonsense |
| *ABCG8* | Chr2 | + | 43873831 | Exon 9 | c.1256T>A | p.I419N | VUS | Missense |
| *ABCG8* | Chr2 | + | 43873842 | Exon 9 | c.1267G>A | p.E423K | VUS | Missense |
| *ABCG8* | Chr2 | + | 43873844 | Exon 9 | c.1269G>T | p.E423D | VUS | Missense |
| *ABCG8* | Chr2 | + | 43873860 | Exon 9 | c.1285A>C | p.M429L | VUS | Missense |
| *ABCG8* | Chr2 | + | 43873980 | Exon 9 | c.1405_1406del | p.S469Qfs*12 | Pathogenic | Frameshift |
| *ABCG8* | Chr2 | + | 43874413 | Exon 10 | c.1418C>G | p.S473X | Likely Pathogenic | Nonsense |
| *ABCG8* | Chr2 | + | 43874432 | Exon 10 | c.1437C>G | p.Y479X | Likely pathogenic | Nonsense |
| *ABCG8* | Chr2 | + | 43874439 | Exon 10 | c.1444del | p.L482Wfs*40 | Pathogenic | Frameshift |
| *ABCG8* | Chr2 | + | 43874471 | Exon 10 | c.1476T>A | p.Y492X | Likely Pathogenic | Missense |
| *ABCG8* | Chr2 | + | 43874482 | Exon 10 | c.1487insA | p.I497Dfs*105 | Likely Pathogenic | Frameshift |
| *ABCG8* | Chr2 | + | 43875157 | Exon 11 | g.41193-202 ins | p.E500DfsX604 | Pathogenic | Frameshift |
| *ABCG8* | Chr2 | + | 43875159 | Exon 11 | c.1502T>C | p.L501P | VUS | Missense |
| *ABCG8* | Chr2 | + | 43875185 | Exon 11 | c.1528_1530delATC | p.I510del | VUS | Frameshift |
| *ABCG8* | Chr2 | + | 43875191 | Exon 11 | c.1534G>A | p.G512R | VUS | Missense |
| *ABCG8* | Chr2 | + | 43875265 | Exon 11 | c.1608G>A | p.W536X | Likely Pathogenic | Nonsense |
| *ABCG8* | Chr2 | + | 43875286 | Exon 11 | c.1629G>C | p.R543S | VUS | Missense |
| *ABCG8* | Chr2 | + | 43875297 | Exon 11 | c.1640T>C | p.L547P | VUS | Missense |
| *ABCG8* | Chr2 | + | 43875366 | Exon 11 | c.1709_1711del | p.F570del | Likely Pathogenic | Frameshift |
| *ABCG8* | Chr2 | + | 43875377 | Exon 11 | c.1715T>C | p.L572P | VUS | Missense |
| *ABCG8* | Chr2 | + | 43875377 | Exon 11 | c.1720G>A | p.G574R | Pathogenic | Missense |
| *ABCG8* | Chr2 | + | 43875378 | Exon 11 | c.1721G>A | p.G574E | Likely Pathogenic | Missense |
| *ABCG8* | Chr2 | + | 43875381 | Exon 11 | c.1724G>A | p.G575D | VUS | Missense |
| *ABCG8* | Chr2 | + | 43875408 | Exon 11 | c.1751G>A | p.W584X | Likely pathogenic | Nonsense |
| *ABCG8* | Chr2 | + | 43875409 | Exon 11 | c.1752G>A | p.W584X | Pathogenic | Nonsense |
| **Gene** | **Chromosome** | **Strand** | **Location** | **Exon/Intron number** | **Nucleotide change** | **Effect of protein** | **ACMG** | **Mutation type** |
| *ABCG8* | Chr2 | + | 43875415 | Intron 11 | c.1756+2T>A |  | Pathogenic | Splicing |
| *ABCG8* | Chr2 | + | 43877566 | Exon 12 | c.1762G>C | p.A588P | VUS | Missense |
| *ABCG8* | Chr2 | + | 43877591 | Exon 12 | c.1787T>G | p.L596R | VUS | Missense |
| *ABCG8* | Chr2 | + | 43877645 | Exon 12 | g.43683-727del | p.M614-K628del | Pathogenic | Frameshift |
| *ABCG8* | Chr2 | + | 43877681 | Exon 12 | c.1877G>T | p.G626V | VUS | Missense |
| *ABCG8* | Chr2 | + | 43877814 | Exon 13 | g.43866-7indelsT | p.V642SfsX662 | Pathogenic | Frameshift |
| *ABCG8* | Chr2 | + | 43877796 | Exon 13 | c.1906del | p.D636fs | Likely pathogenic | Frameshift |
| *ABCG8* | Chr2 | + | 43877840 | Exon 13 | c.1949T>G | p.L650R | VUS | Missense |
| *ABCG8* | Chr2 | + | 43877865 | Exon 13 | c.1974C>A | p.Y658X | Likely Pathogenic | Nonsense |
